# Cranial anatomy of the gorgonopsian *Cynariops robustus* based on CT-reconstruction

**DOI:** 10.1371/journal.pone.0207367

**Published:** 2018-11-28

**Authors:** Eva-Maria Bendel, Christian F. Kammerer, Nikolay Kardjilov, Vincent Fernandez, Jörg Fröbisch

**Affiliations:** 1 Museum für Naturkunde, Leibniz-Institut für Evolutions- und Biodiversitätsforschung, Berlin, Germany; 2 North Carolina Museum of Natural Sciences, Raleigh, NC, United States of America; 3 Evolutionary Studies Institute & School of Geosciences, University of the Witwatersrand, Johannesburg, South Africa; 4 Helmholtz-Zentrum Berlin–Institute of Applied Materials, Berlin, Germany; 5 Imaging and Analysis Centre, The Natural History Museum, London, United Kingdom; 6 European Synchrotron Radiation Facility, Grenoble, France; 7 Institut für Biologie, Humboldt-Universität zu Berlin, Berlin, Germany; Monash University, AUSTRALIA

## Abstract

Gorgonopsia is one of the major clades of non-mammalian synapsids, and includes an array of large-bodied carnivores that were the top terrestrial predators of the late Permian. Most research on the clade has focused on these largest members; small-bodied gorgonopsians are relatively little-studied. Here, we redescribe a small gorgonopsian skull (MB.R.999) from the late Permian (*Tropidostoma* Assemblage Zone) of South Africa on the basis of neutron and synchrotron CT reconstructions, which yield new data on internal cranial morphology in Gorgonopsia. Because of the largely undistorted nature of MB.R.999, we were also able to reconstruct unossified areas such as the brain endocast and the otic labyrinth. MB.R.999 can be referred to the taxon *Cynariops robustus* based on its general skull proportions, postcanine tooth count, preparietal morphology, and vomerine morphology. We refer additional small gorgonopsian specimens from the Victoria West area to *Cynariops robustus*, and consider *Cynarioides grimbeeki* and *Cynarioides laticeps* to be synonymous with *C*. *robustus*. Inclusion of *Cynariops* in a phylogenetic analysis of Gorgonopsia recovers it within a large clade of African taxa, more closely related to *Lycaenops* and rubidgeines than *Eriphostoma* or *Gorgonops*.

## Introduction

The therapsid clade Gorgonopsia is best known for including the apex predators of the late Permian: bear-sized (up to 60 cm skull length), saber-toothed taxa such as *Inostrancevia* and *Rubidgea* [1–3]. Taxa such as these represent only a small fraction of gorgonopsian diversity, however. Although all known gorgonopsians are inferred to have been predators, they spanned a wide range of sizes and included numerous smaller species in addition to the aforementioned giants. Indeed, the earliest known gorgonopsians (from the middle Permian) are all relatively small animals, with skull lengths in the 10–15 cm range [4]. Although gorgonopsians began to greatly exceed this size during their diversification in the late Permian, small-bodied gorgonopsians continued to make up a major portion of the clade’s diversity and abundance right up until its extinction at the end of the Permian [4].

The anatomy of smaller gorgonopsian taxa has been poorly studied compared with their larger relatives. Owen [5] provided a reasonably thorough description of the small (total skull length estimated 10–12 cm) gorgonopsian *Aelurosaurus felinus*, but was limited by the preparation available at the time and the incompleteness of the type specimen (NHMUK PV R339). The majority of subsequent gorgonopsian descriptions, from the early 20^th^ century, were more cursory, generally focusing on only a few features (e.g. tooth count, preparietal shape) believed to be of taxonomic importance [6–9]. Notable exceptions in this regard are the monographs of Pravoslavlev [10] and Colbert [11]. However, while these works included comparisons with some of the small-bodied gorgonopsians, they focused on larger taxa (*Inostrancevia* and *Lycaenops*, respectively). The same can generally be said for more recent gorgonopsian scholarship. Among the few thorough modern descriptions of gorgonopsian cranial anatomy, most have focused on large (skull length >30 cm) (e.g. *Sycosaurus* [2, 12]) or mid-sized (skull length 20–30 cm) taxa (e.g. *Arctops*, *Gorgonops* [4, 13, 14]).

Only a few recent papers deal extensively with cranial morphology in small (10–15 cm skull length) gorgonopsians. Ivakhnenko [15] presented a thorough description of the skull of the Russian gorgonopsian *Suchogorgon golubevi* based on a series of partially disarticulated skulls. Kammerer [16] redescribed the middle Permian South African gorgonopsian *Eriphostoma microdon* based on computed tomographic (CT) scans of the holotype and later [17] on the basis of newly-discovered referable material. Most recently, Araújo et al. [18] used CT data to describe the basicranial anatomy and neuroanatomy of a small gorgonopsian specimen (GPIT/RE/7124) historically referred to *Aloposaurus gracilis* [19]. CT-scanning represents a powerful and non-destructive tool for the study of small gorgonopsians, including the many taxa based on poorly-prepared holotypes, and we recommend its broader use in the study of this clade.

Here, we redescribe a historically-collected small gorgonopsian specimen, MB.R.999, based on CT data. This specimen was initially described by Janensch [20], but his description was limited to surface details of the skull and lower jaw. The taxonomically-important anterior palate of MB.R.999 is unprepared, and an understanding of the morphology of the vomer and palatal bosses has only become possible based on scans. Additionally, scanning of the specimen has permitted description of the internal surfaces of the various skull and jaw elements, previously not possible for this specimen and at present described in few gorgonopsians of any kind.

## Material and methods

**Fig 1:**
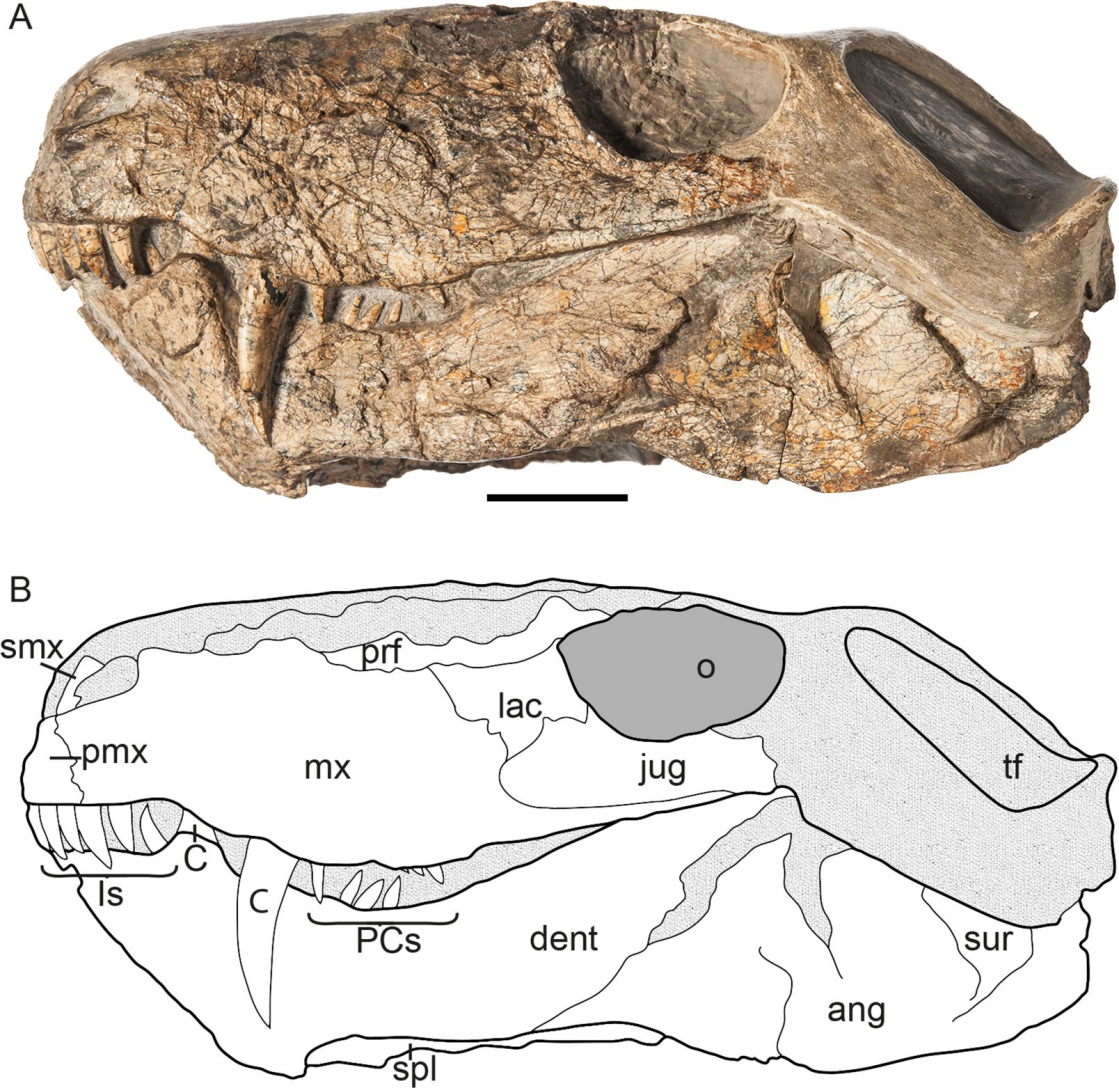
**MB.R.999, (A) photograph (reprinted [22] under a CC BY license, with permission from Carola Radke, MfN, original copyright 2016) and (B) interpretative drawing of specimen in left lateral view.** Abbreviations: ang, angular; C, canine; dt, dentary; Is, incisors; jug, jugal; lac, lacrimal; max, maxilla; o, orbit; PCs, postcanines; pmx, premaxilla; prf, prefrontal; smx; septomaxilla; spl, splenial; sur, surangular. Scale bar = 2 cm.

**Fig 2:**
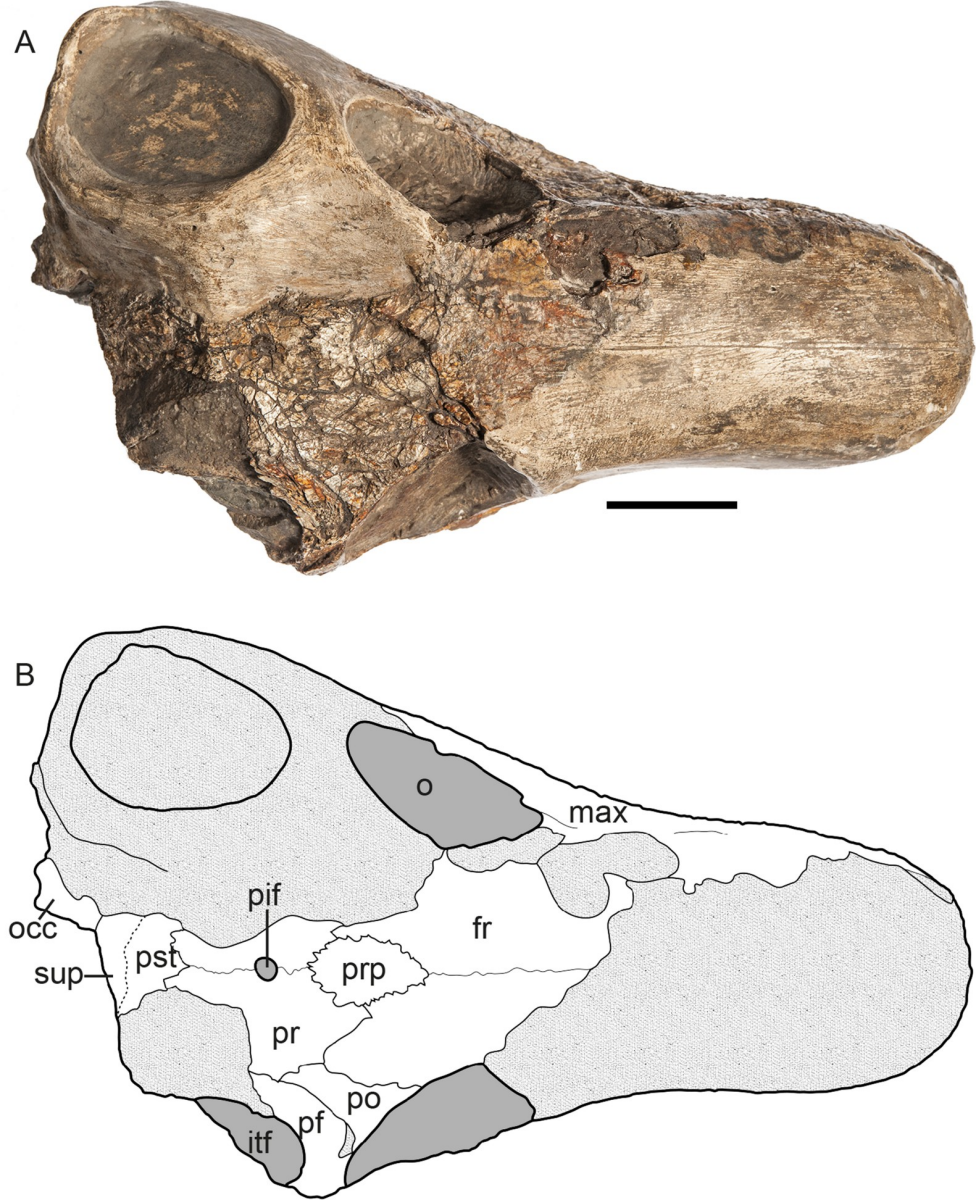
**MB.R.999, (A) photograph (reprinted [22] under a CC BY license, with permission from Carola Radke, MfN, original copyright 2016) and (B) interpretative drawing of specimen in dorsal view.** Abbreviations: fr, frontal; max, maxilla; o, orbit; occ, occiput; pf, postfrontal; pif, parietal foramen; po, postorbital; pr, parietal; prp; preparietal; pst, postparietal; sup, supraoccipital, tf, temporal fenestra. Scale bar = 2 cm.

**Fig 3:**
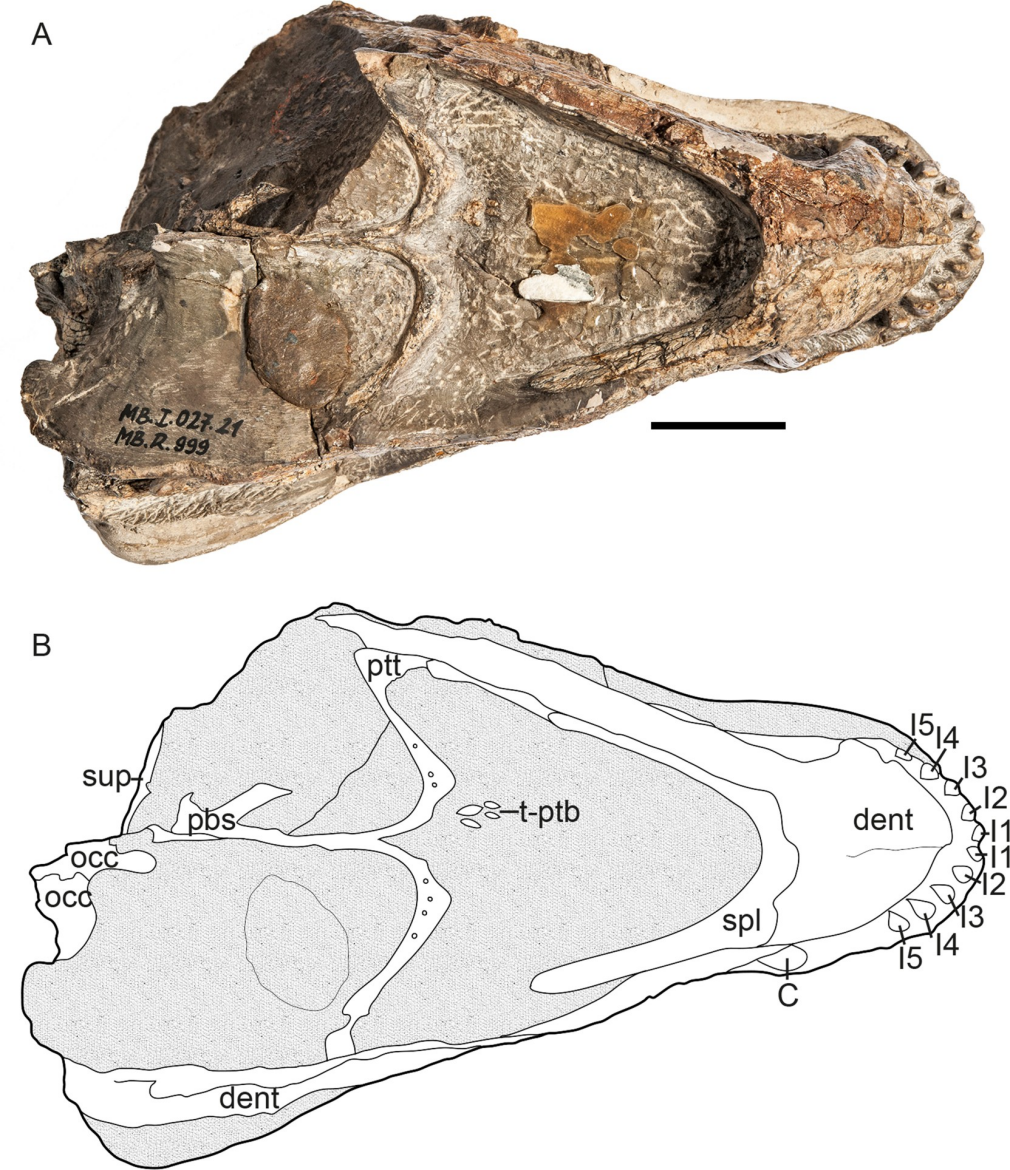
**MB.R.999, (A) photograph (reprinted [22] under a CC BY license, with permission from Carola Radke, MfN, original copyright 2016) and (B) interpretative drawing of specimen in ventral view.** Abbreviations: C, canine; dt, dentary; I1-I5, incisors 1–5; occ, occiput; pbs, parabasisphenoid; ptt, transverse process of the pterygoid; sup, supraoccipital; t-ptb, teeth on the pterygoidal boss. Scale bar = 2 cm.

The specimen described herein (MB.R.999, Figs 1–3), was labeled in the catalogue of the Museum für Naturkunde, Berlin (S1 Fig), as a specimen of the gorgonopsian genus *Aelurognathus*, and previously referred to in the literature as *Aloposaurus*? sp. by Janensch [20]. This specimen represents a cranium and associated mandible. It is undistorted, but several portions of the skull have been lost to erosion. The dorsal portion of the snout and the left temporal region are missing and have been restored in plaster. Parts of the right posterior portion of the skull and mandible are also currently missing, but were originally preserved with this specimen, as they were figured by Janensch [20]. MB.R.999 was collected by Janensch at the locality Biesiespoort (Northern Cape Province, South Africa) in 1929 and later prepared by E. Siegert and J. Schober. The following description of the specimen is based on personal examination by the authors and CT reconstruction by EMB. Neutron tomographic scanning was performed by NK, synchrotron scanning by VF. Comparisons with other gorgonopsian specimens were based on literature (primarily [3, 4, 15, 21]), specimen photographs from CFK, and personal examination of specimens by EMB and CFK. Specimen photographs of MB.R.999 shown in Figs 1–3 were taken by Carola Radke (photographer at the Museum für Naturkunde, Berlin), except for those highlighting the teeth (taken by EMB) as well as the type and referred material of *Cynariops robustus* (taken by CFK). No permits were required for the described study, which complied with all relevant regulations.

### Computed tomography

The specimen MB.R.999 was scanned at two different facilities using distinct techniques, due to varying results in contrast and resolution in each scan. Data received from synchrotron scans provided more detail regarding the specimen’s dentition (better resolution), whereas neutron tomography showed substantially clearer sutural details (better contrast). Visualization of the data was performed in VG Studio Max 2.2 by EMB.

*Synchrotron*.—MB.R.999 was analyzed using Propagation Phase Contrast Synchrotron Radiation micro-Computed Tomography (PPC-SR-μCT) at the BM05 beamline of the European Synchrotron Radiation Facility (ESRF, Grenoble France) using filtered white beam (Al: 6 mm, Cu: 6mm) with a total integrated energy of about 129 keV. The sample-detector distance was about 4.2m to observe sufficient phase contrast effect by free-air propagation.

The optic setup consisted of Scintillating optical fibers, a x 0.3 magnification set of lenses and a FReLoN-2k camera, resulting in a measured 45.48 μm isotropic pixel size on the recorded radiographs. To compensate for the noise of the camera, 3000 projections were recorded over 360°, with an exposure time of 80 ms each (as well as 21 flat field images and 20 dark field images). The center of rotation was shifted by 200 pixels to increase the final horizontal field of view in the final tomograms (so-called half-acquisition protocol). Given vertical size of the incident beam (~3.6 mm), 60 scans were needed to cover the full length of the skull, with a vertical displacement of 2.5 mm between two consecutive scans (30% of overlap to compensate for the vertical intensity profile of the X-ray beam). Tomographic reconstruction was performed using filtered-back projection from the PyHST2 software with Paganin algorithm for single distance phase retrieval [23, 24].

Due to the presence of a mineral phase substantially denser than the surrounding sediment, the classic Paganin algorithm was not appropriate for the fossil or the denser mineral. Because of this, the multi-phase approach of the PyHST2 software was used: a first reconstruction was done with parameters adapted to the denser material (δ/β = 1000); in the resulting reconstructed volume, threshold segmentation permitted isolation of this dense part (threshold at 0.47 on the 32 bits float data). The segmentation mask was then imported in a classic filtered-back projection reconstruction to isolate the denser phase. In the next step, a set of radiographs containing only the segmented phase was generated. The information from this new set of data was subtracted from the original set of radiographs. The altered radiographs were used to reconstruct the volume with Paganin parameters adapted this time to the less dense material (fossilized bone and sediment, δ/β = 2000). Ultimately, this last reconstruction and the segmented part of dense material from the first reconstruction were combined to produce a final volume with Paganin parameters adapted to the two main density phases of the sample.

The generated 32-bit volumes were converted to 16-bit stacks of TIFF (using min and max 32-bit crop values from the 3D histogram provided by PyHST2). For the final vertical concatenations of the series of scans, advantage of the important overlap between consecutive scans has been taken and a weighted average of similar slices was performed, giving more weight to a slice when it was closer to the center of the stack than to the border (as the intensity of the X-ray beam is greater at the center). Ring artefacts were corrected on reconstructed slices using an ESRF in-house Matlab script [25]. Finally, a 2x2x2 binning was applied to the final set of data to generate a version easier to handle.

The files received from the ESRF were reverse mirrored along the longitudinal axis at the μCT-Laboratory of the Museum für Naturkunde—Leibniz Institute for Evolution and Biodiversity Science in ImageJ [26] to reflect the specimen’s original orientation and subsequently visualized in VG Studio Max 2.2 by EMB.

*Neutron tomography*.*—*The neutron tomography instrument CONRAD (Cold Neutron Tomography and Radiography) at the Helmholtz Centre Berlin for Materials and Energy, Germany, is located at a curved neutron guide at the BER-II research reactor [27]. The curved guide closes the direct view to the reactor core and acts as a filter, which eliminates the high energetic neutrons and gammas from the core. In this way the neutron spectrum at the end of the guide has a maximum at 2.5 Å—the so-called ‘cold’ neutrons—which show higher sensitivity to hydrogen and other light elements like lithium and boron. The facility uses a pinhole geometry with variable diameters, D, at the end of the guide (D = 1, 2 or 3 cm) and a fixed flight path between the pinhole and the detector of L = 10 m. For the experiment presented in this study, a pinhole diameter of 3 cm was used resulting in a collimating ratio L/D of 330, respectively. As a detector system, a setup with a pixel size of 100 μm and Field-of-View of 200 mm x 200 mm was used [28]. Exposure time was 45 s per projection. The tomography experiment was performed with 600 projections reflecting a total measuring time of 9 hours. Effective voxel size was 0.1 mm. 360° parallel beam reconstruction was performed using Octopus Reconstruction software, version 8.8.2.7 [29]. A 120 noise filter and beam hardening correction of 0.2 were applied. Specimen reconstruction based on this data was done in VG Studio Max 2.2 by EMB.

### Institutional Abbreviations

AMNH FARB, American Museum of Natural History, Fossil Amphibian, Reptile, and Bird Collection, New York, USA; BP, Evolutionary Studies Institute (formerly the Bernard Price Institute for Palaeontological Research), University of the Witwatersrand, Johannesburg, South Africa; GPIT, Paläontologische Sammlung, Eberhard Karls Universität Tübingen, Germany; MB.R., Museum für Naturkunde, Fossil Reptile Collection, Berlin, Germany; NHMUK PV, Natural History Museum, Vertebrate Palaeontological Collection, London, UK; UCMP, University of California Museum of Paleontology, Berkeley, USA; UMZC, University Museum of Zoology, Cambridge, UK; SAM, Iziko Museums of South Africa, Cape Town, South Africa; TM, Ditsong, the National Museum of Natural History (formerly the Transvaal Museum), Pretoria, South Africa.

## Results

### Description

Although it is missing portions of the snout and temporal arches, the intact portions of MB.R.999 are generally well preserved, in spite of some surficial cracking on the left maxilla and intertemporal region. The specimen is essentially undistorted (Figs 1–3). Eroded portions of the skull have been reconstructed with plaster, obscuring the underlying morphology. The posteriormost right side of the skull, including much of the squamosal, a large part of the braincase, and the right postdentary elements of the jaw, is missing. This fragment had been fully prepared and is figured by Janensch [20], but could not be located in a recent search of the MB.R. collections.

The skull is 15.5 cm long (basal length, also see Table 1) and 6.0 cm wide between the lateral tips of the transverse processes of the pterygoid (maximum width of skull cannot be confidently measured, because of damage to the temporal arcade). The occiput is approximately 5.5 cm in height.

**Table 1:** General measurements of specimen MB.R.999.

*Basal skull length (premaxilla to occipital condyle)*:	*15*.*5 cm*
*Snout length (premaxilla to anterior rim of orbit)*:	*8*.*2 cm*
*Orbit height (left side)*:	*2*.*5 cm*
*Minimum interorbital width*:	*3*.*1 cm*
*Length of vomerine process of premaxilla*	*1*.*2 cm*
*Length of expanded body of vomer*	*2*.*6 cm*
*Length of basicranial girder*	*5*.*7 cm*
*Height of dentary symphysis*	*3*.*0 cm*
*Height of dentary ramus*	*1*.*9 cm*

#### Cranium

**Fig 4:**
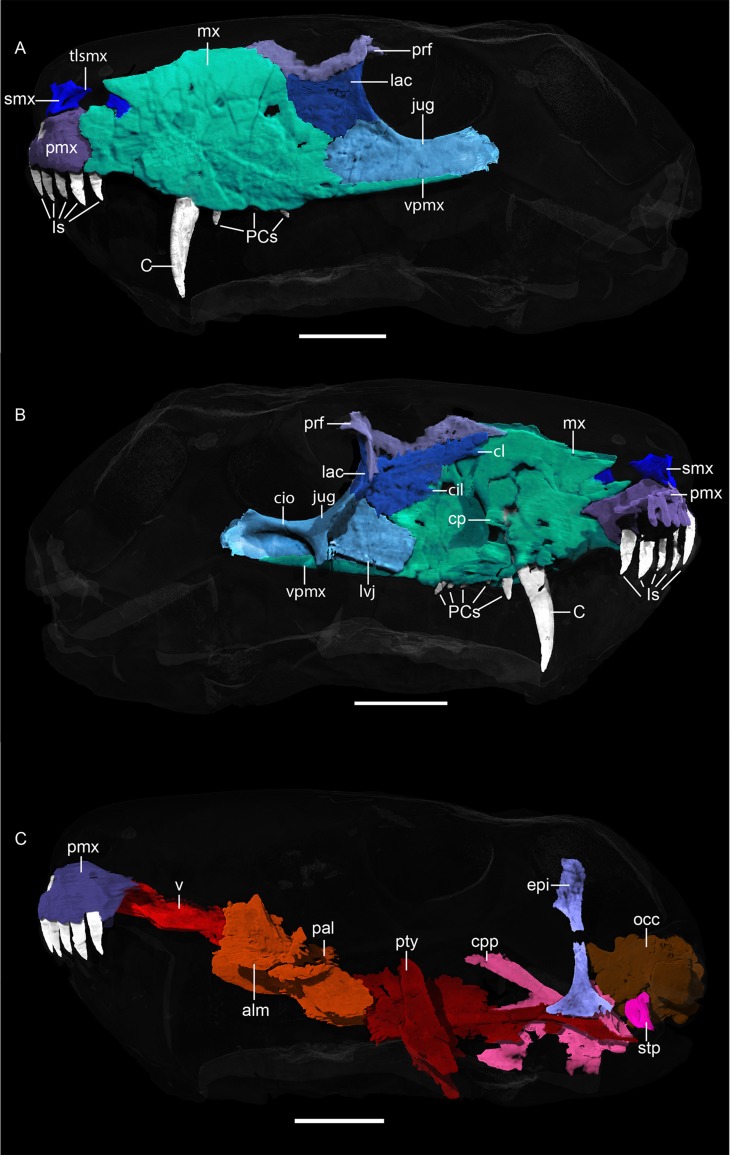
**Neutron CT reconstruction of MB.R.999: (A) left lateral view and (B) left medial view of the face, (C) palatal morphology from left lateral.** Abbreviations: alm, ala maxillaris of palatine; C, canine; cpp, cultriform process of parabasisphenoid; epi, epipterygoid; cil, crista infralacrimalis; cio, crista infraorbitalis; cl, crista lacrimalis; cp: canine-bearing protuberance; Is, incisors; jug, jugal; lac, lacrimal; lvj, lamina ventralis of the jugal; mx, maxilla; occ, occiput; pal, palatine; pbs, parabasisphenoid; PCs, postcanines; pmx, premaxilla; prf, prefrontal; pty, pterygoid; smx; septomaxilla; stp, stapes; tlsmx, transverse lamina of the septomaxilla; v, vomer; vpmx; ventral process of the maxilla. Scale bars = 2 cm.

**Fig 5:**
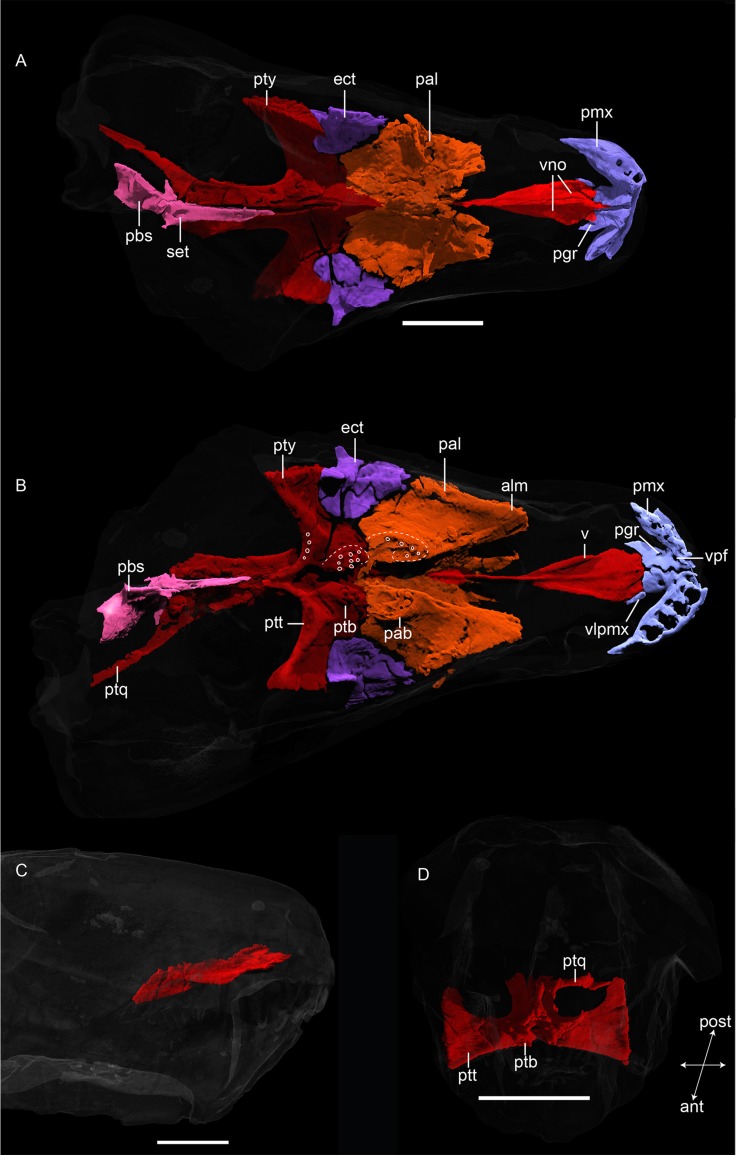
**Neutron CT reconstruction of MB.R.999: (A) dorsal and (B) ventral palatal morphology, (C) vomer from right lateral, (D) pterygoid from anterior.** White circles in (B) indicate position of teeth. Abbreviations: ect, ectopterygoid; pab, bosses of the palatine; pal, palatine; pbs, parabasisphenoid; pgr, premaxillary groove; pmx, premaxilla, ptb, bosses of the pterygoid; ptq, quadrate ramus of the pterygoid; ptt, transverse process of the pterygoid; pty, pterygoid; set, sella turcica; v, vomer; vno, hypothetical position of vomeronasal organ; vpf: ventral premaxillary foramen; vlpmx, ventral lamina of the premaxilla. Scale bars = 2 cm.

*Premaxilla*.*—* Only the palatal and lateral subnarial portion of the premaxilla is preserved (Figs 4, 5A and 5B). It is arcuate in ventral view and houses five incisors and the same number of replacement teeth on each side. Posteriorly, its lateral surface is partially covered by the maxilla. The internarial portion of the premaxilla is broken off. The naris is not completely preserved but its ventral margin is made up in part by the premaxilla. The palatal portion of the premaxilla exhibits a deep invagination between its posterolateral border and the long vomerine process medially, forming the anterior edges of the choana. The vomerine process is a flattened, posteriorly-directed splint of bone that contacts the broad anterior border of the vomer and surrounds it laterally. Ventrally, a distinctly interdigitated median suture is present between the vomerine processes (Fig 5B); dorsally, this suture is relatively straight (Fig 5A). Two pairs of openings are visible on the ventral surface of the premaxillae. Anteriorly, a rounded foramen is present behind the alveolus for the first incisor, near the contact between the main body of the premaxilla and the vomerine process. This ventral premaxillary foramen would have housed blood vessels [13]. Posteriorly, on the vomerine process, a second, usually somewhat mediolaterally-angled opening is present, which Kammerer [13] termed the premaxillary groove. In MB.R.999 the left groove is circular. Kammerer [13] argued that these grooves represent ducts for the vomeronasal organs, which would have been situated on the dorsal surface of the vomer in early therapsids [30]. In MB.R.999, these grooves do appear to perforate the vomerine process and open dorsally at the anterior tip of an elongate depression on the vomer that would house the vomeronasal organ (Fig 5A).

**Fig 6:**
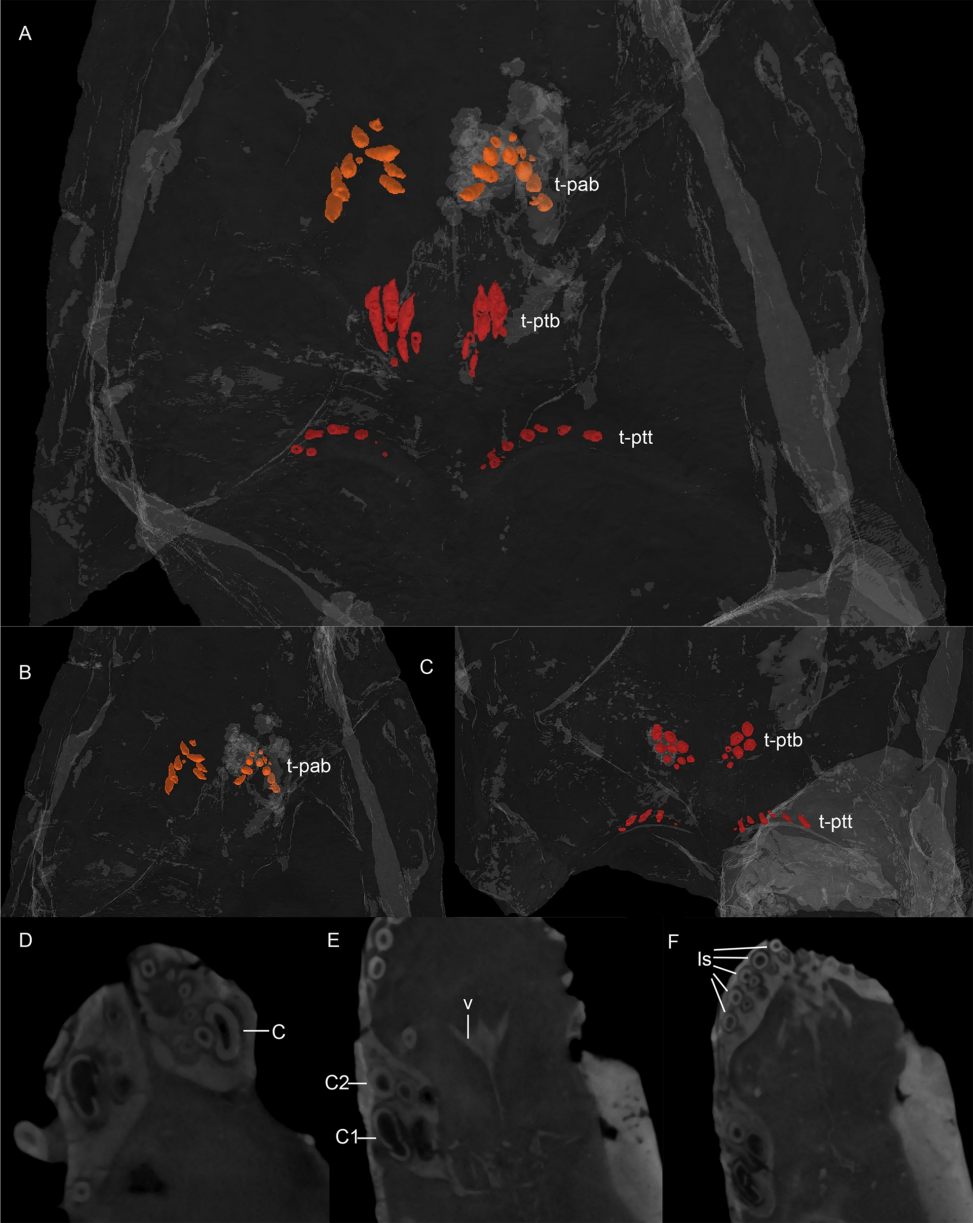
MB.R.999: Teeth and their replacements. (A) all palatal teeth, Synchrotron CT, (B) teeth on palatine, snout slightly tilted to dorsal, Synchrotron CT, (C) teeth on pterygoid, snout slightly tilted to ventral, Synchrotron CT, (D) canine of right lower jaw and replacements lingually to it, 2D slice of Neutron CT, (E) canine of left upper jaw and its replacements mesially and lingually to it, 2D slice of Neutron CT, (F) incisors and their replacements (lingually to them) in the left upper jaw, 2D slice of Neutron CT. Abbreviations: C, canine; C1, functional canine; C2, second but non-functinal canine; Is, incisors; t-pab, teeth on the bosses of the palatine; t-ptb, teeth on the bosses of the pterygoid; t-ptt, teeth on the transverse processes of the pterygoid; v, vomer.

**Fig 7:**
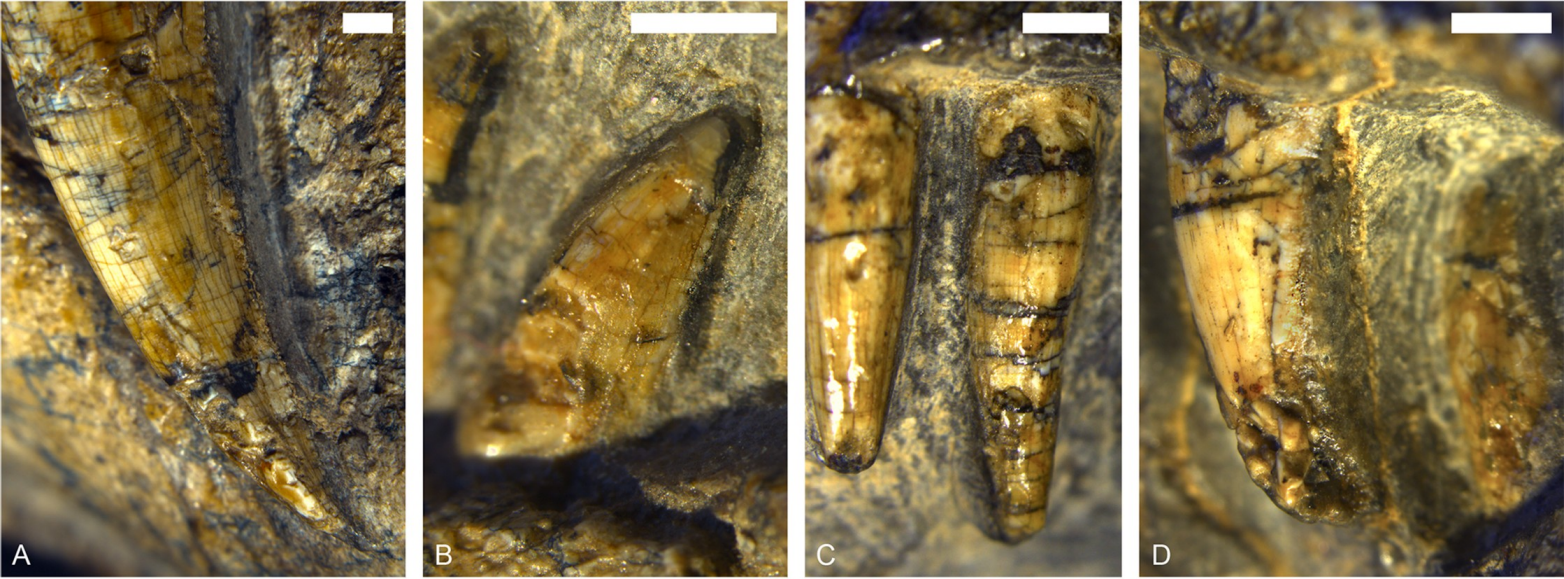
Dentition of MB.R.999. (A) left upper canine, (B) third lower postcanine, (C) first and second upper incisors, (D) first upper incisor.

The upper incisors and their replacements (Figs 4 and 6F) are circular in digital cross-section. The second and fourth of the five incisors are slightly longer than the first, third and fifth (~0.9 cm vs. ~0.65 cm), which probably reflects replacement history rather than true irregularity in size along the tooth row. It is unclear if a serrated distal carina is present on the incisors (Fig 7D) because this part is concealed by matrix. The surface enamel on the incisors is smooth, though weak longitudinal bands are visible on their outer surface (Fig 7C), giving the appearance of striations.

*Septomaxilla*. *—* Two portions of the left septomaxilla are preserved (Fig 4A and 4B). The anterior one sits dorsally on the premaxilla and constitutes a dorsal process that expands into a transverse lamina within the naris (this lamina, which separates the naris into dorsal and ventral compartments, is considered a synapomorphy of Gorgonopsia [2]). This process is also broad ventrally and trapezoidal in cross section. The other preserved portion of the septomaxilla is a thin lamina inserting into the anterior border of the maxilla. Typically in therapsids the posterior process of the septomaxilla extends between the maxilla and nasal [31], but terminal insertion into the maxilla alone is also known in some other gorgonopsians (e.g. *Viatkogorgon* [32]).

*Nasal*. *—* The nasals are not preserved in MB.R.999.

**Fig 8:**
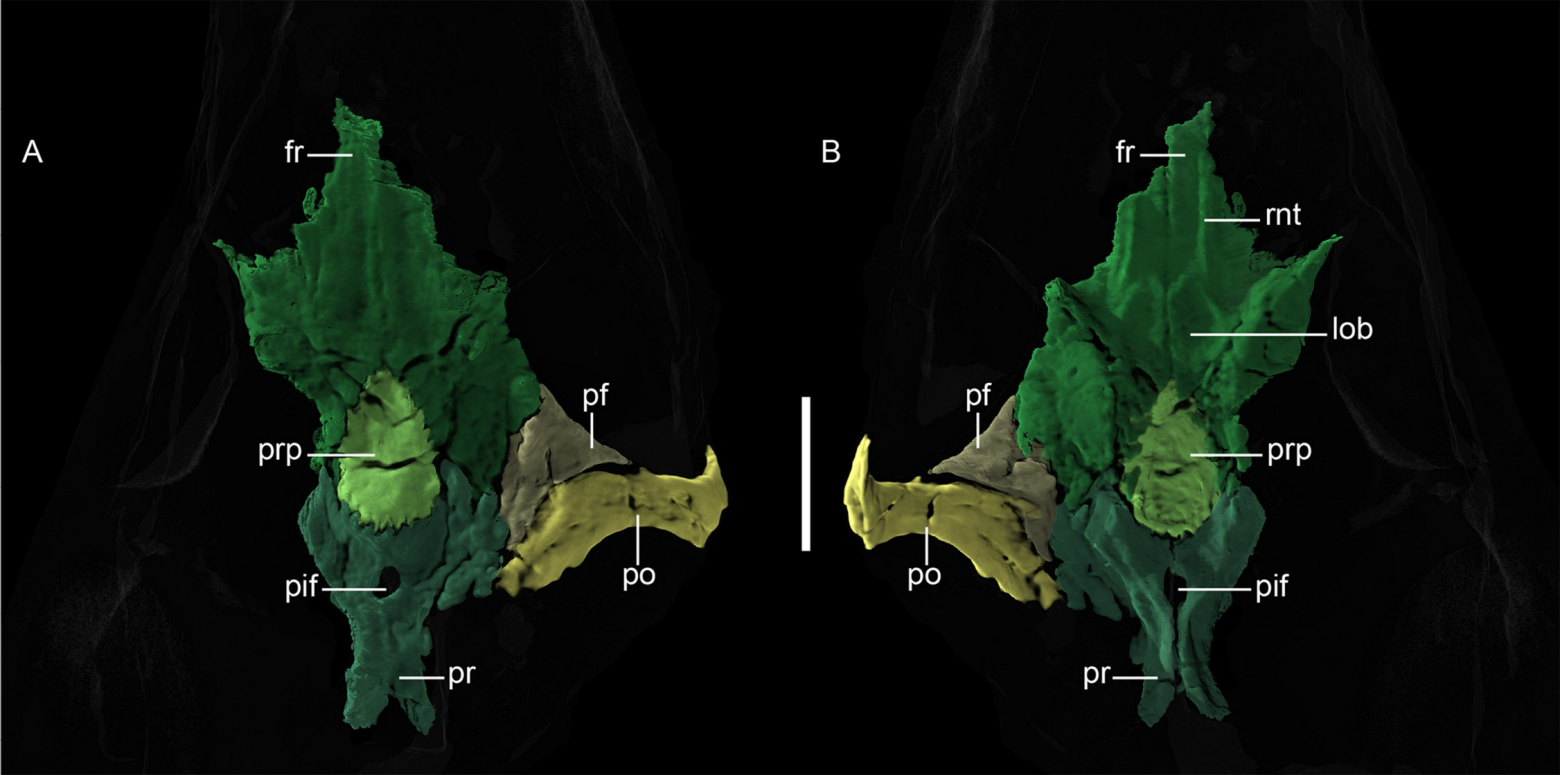
**Neutron CT reconstruction of MB.R.999: skull roof from (A) dorsal and (B) ventral.** Abbreviations: fr, frontal; lob, (position of) left olfactory bulb; pf; postfrontal; pif, parietal foramen; po, postorbital; pr, parietal; prp, preparietal; rnt: ridge for cartilaginous nasal turbinals. Scale bars = 2 cm.

**Fig 9:**
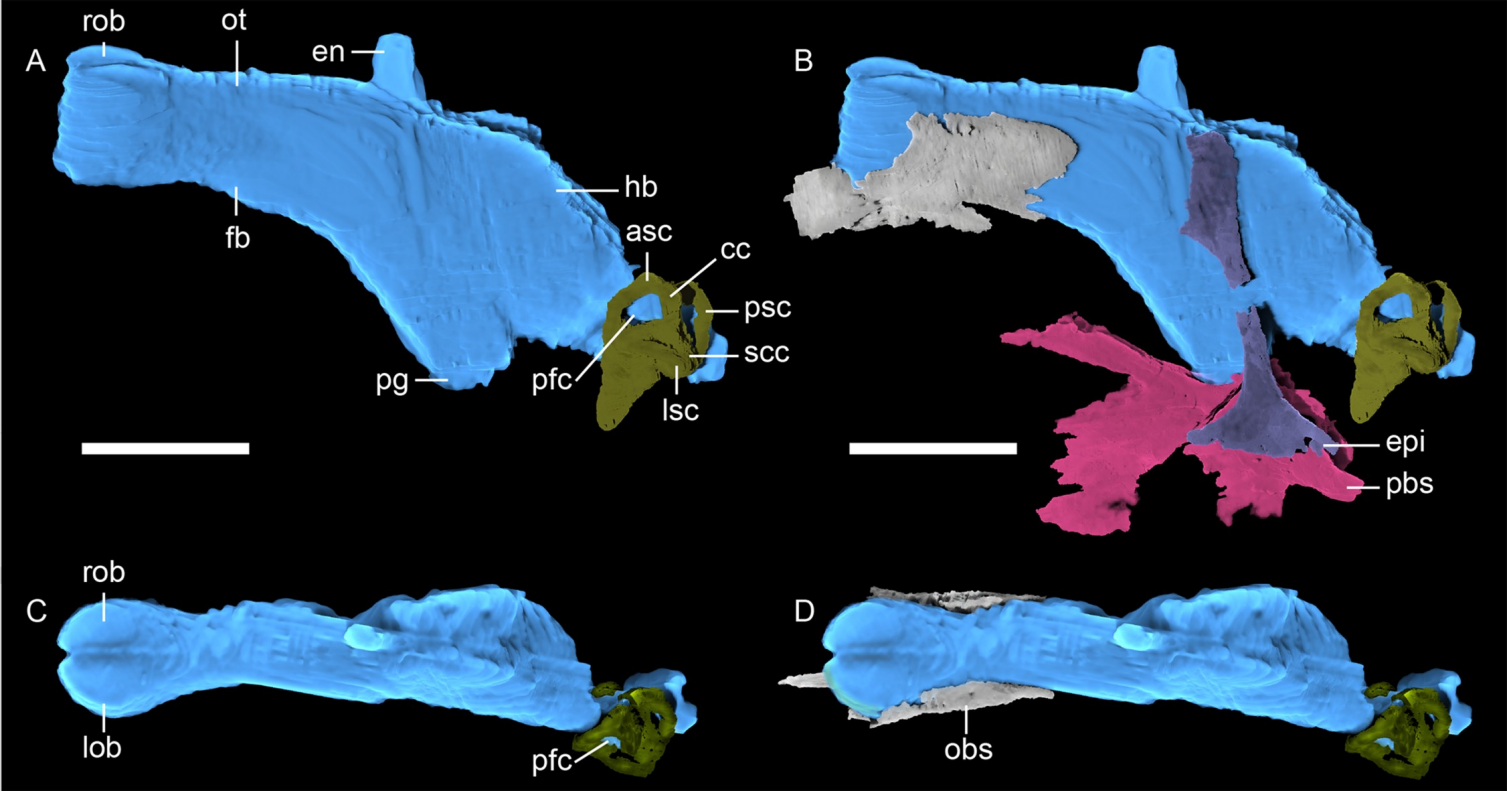
**Neutron CT reconstruction of MB.R.999: brain endocast from (A and B) left lateral and (C and D) dorsal.** Abbreviations: asc, anterior semicircular canal; cc, common crus; en, epiphyseal nerve; epi, epipterygoid; fb, forebrain; hb, hindbrain; lob, left olfactory bulb; ot, olfactory tract; pbs, parabasisphenoid; pfc, paraflocculus; pg, pituitary gland; psc, posterior semicircular canal; rob, right olfactory bulb; scc, secondary common crus. Scale bars = 2 cm.

*Frontal*. *—* The frontal (Fig 8) makes up the largest part of the preserved skull roof of MB.R.999 and is a mostly flat element. Both frontals are damaged: the left frontal is missing a large posterior portion and the right frontal is missing its anterolateral tip. The midline suture between the paired frontals can be identified as a thin ridge along the dorsal surface. The frontal has a broad contribution to the dorsal orbital margin. An interdigitating suture is present between the frontals and the preparietal, whereas their sutures with the parietals and the postfrontal are smooth. Due to the absence of the nasals in MB.R.999, the morphology of the naso-frontal suture is unknown. A series of ridges and depressions are present on the ventral surface of the frontal (Fig 8B): A paired oval depression anterior to its contact with the preparietal, which would have constituted the dorsal silhouettes of the olfactory bulbs (Fig 9). Slightly lateral to the acute anterior demarcation of these depressions, two parallel longitudinal ridges run anteriorly. They are assumed to correspond with the cartilaginous nasal turbinals and would have continued onto the nasals [12, 33].

*Preparietal*. *—* The preparietal (Fig 8) is an unpaired bone located along the cranial midline between the frontals and parietals. In MB.R.999, it is ‘arrowhead’-shaped, with a pointed anterior end and broader posterior margin. The acuminate, interdigitated anterior margin extends between two posterior processes of the frontals. Posteriorly, the preparietal also has an interdigitated suture with the parietals. The preparietal in MB.R.999 is a relatively large element, proportionally similar to that of *Eriphostoma* [17]. Its ventral surface is slightly concave and rugose, not smooth as described by Ivakhnenko [15] for *Suchogorgon*.

*Parietal*. *—* The paired parietals (Fig 8) are dorsally flattened elements that are anteroposteriorly longer than wide. Broad, somewhat curving anterior processes of the parietals frame the posterior half of the preparietal. Although incomplete, it is clear that the parietal made up a large portion of the intertemporal region of MB.R.999, as is typical of gorgonopsians [2, 3]. The midline suture between the parietals is perforated by a large pineal foramen, which does not contact the preparietal. The posterior border of the parietals has an interdigitated suture with the postparietal. The ventral surface of the parietal bears a well-developed ridge that flanks the mid-parietal suture posteriorly before curving anterolaterally around the pineal foramen and terminating near the contact with the frontal. The area between these ridges where they diverge anteriorly is concave and constitutes a posterior extension of the ventral depression on the preparietal.

*Maxilla*. *—* The maxilla typically makes up the largest part of the lateral side of the snout (Fig 4A and 4B) in gorgonopsians. Only the left maxilla is preserved completely in MB.R.999. Anteriorly, a thin maxillary lamina overlies the premaxilla, such that the base of the fifth incisor appears to be beneath the maxilla in lateral view (Fig 4A). The internal surface of the maxilla is smooth anteriorly and its dominant feature is a bulbous protuberance around the root of the canine. Posterior to the canine root, the medial surface of the maxilla is more rugose. Like the incisors, the canine is only serrated on its distal margin. The subrectangular denticles forming the serrated edge of the canine are larger and compared to those on the postcanines (2 per 1mm and 4 per 1 mm, respectively, Fig 7A and 7B). The canine is oval in cross-section with a medial indentation (Fig 6E). A long diastema (~1.8 cm) between the incisors and the canine is present at the anterior alveolar margin of the maxilla, although this length is exaggerated by the position of the functional canine in the posterior alveolus. The remains of an old, not yet shed or resorbed canine root with the rudiment of a replacement canine are visible in the anterior alveolus in the CT scan (Fig 6E). There is no bony partition separating the anterior and posterior alveoli internally. This condition is similar to that described by Kermack [34] for other small gorgonopsians. The robust, posterodorsally-angled canine root is almost as long as the exposed crown.

Five small, conical postcanines (circular in cross section and with distal serrations) closely follow the canine. One replacement tooth is situated medially to the second postcanine tooth and can only be distinguished from the others because of its position. This also mirrors the description of gorgonopsian dentition by Kermack [34], although *contra* that study we found the maxillary postcanine alveoli to be separated by bony interdental plates. The postcanine tooth row is closely packed, and terminates ~1.5 cm anterior to the anterior margin of the orbit.

Posteriorly, the maxilla extends into an elongate ventral process, which undercuts the jugal before terminating beneath the posterior half of the orbit. Anteriorly, this process merges into the main facial portion of the maxilla in the form of a weak ridge, which produces a slight concavity above the tooth row but not a distinct labial emargination as in *Eriphostoma* or rubidgeines [2, 35]. The maxilla bulges medially internal to this concavity, and ventromedially forms a flat sutural facet for the anterior part of the jugal. A short maxillary process extends between the lacrimal and jugal both externally and internally. Dorsally, the maxilla is broadly rounded and overlaps the anterior margins of the lacrimal and the prefrontal.

*Lacrimal*. *—* The lacrimal (Fig 4A and 4B), of which only the left is preserved in MB.R.999, is roughly quadrilateral in external view. Internally, however, it bears a short, thin anterior process overlapped by the maxilla laterally, which is similar in morphology to a corresponding process of the prefrontal. This process bears a slight longitudinal crest (= crista lacrimalis of Ivakhnenko [15]). A second crest (= crista infralacrimalis of Ivakhnenko [15]) emerges from the origination point of the first one near the orbit but extends horizontally towards the maxilla, onto which it does not continue. As one of the circumorbital bones, the lacrimal forms the orbit’s anterior margin and bears a distinct lacrimal foramen exiting into the orbit. The orbital margin of the lacrimal has a concave surface posteriorly and internally forms a well-developed ridge that originates on the prefrontal and continues posteroventrally onto the jugal.

*Prefrontal*. *—* The prefrontal of MB.R.999 is only partially preserved on the left side (Fig 4A and 4B); it is broken off dorsally. It is not preserved at all on the right side. The missing portion would have formed an anterolateral portion of the interorbital region; the preserved portion forms the anterodorsal margin of the orbit and part of the lateral snout surface. Like the lacrimal, internally the prefrontal extends further anterior than is visible laterally, in the form of a narrow, attenuate process. Posteriorly its contribution to the anterior orbital rim is curved, as in the lacrimal and jugal. The flat element constituting part of the anterior orbital margin is almost laminar. Its most ventral part is situated anterior to the lacrimal so that they overlap (not mentioned either in Ivakhnenko’s [15] or Sigogneau-Russel’s [3, 4] studies on gorgonopsian skull anatomy).

*Postfrontal*. *—* Only the right postfrontal is preserved in MB.R.999, situated between the frontal and postorbital at the posterodorsal corner of the orbit (Fig 8). It is roughly triangular in outline and contributes anteriorly to the orbit, anteromedially borders the frontal, posterolaterally borders the postorbital. Posteromedially it has only a short contact with the parietal. The sutures between the postfrontal and all surrounding bones are generally straight, not interdigitated. Ventrally, a small posterior flat process overlaps the postorbital partially via a corresponding ridge on that element.

*Postorbital*. *—* The right postorbital is incompletely preserved; what is present has the shape of a long, thick band (Fig 8) forming the dorsal part of the postorbital bar and slightly projecting posterodorsally behind the orbit, at the anteromedial corner of the temporal fenestra. Due to surface damage, the trabecular structure of the bone is partially exposed, giving the (false) appearance of pits and ridges running along its mediolateral extension in the CT reconstruction. At the base of the postorbital bar, a slight contact with the jugal is discernible anteriorly. When intact, the base of this bar should take the form of an expanded postorbital footplate, as shown by Janensch [20]. The left postorbital is not present, although the missing part was depicted by Janensch [20].

*Jugal*. *—* Ventral to the lacrimal, the jugal extends posteriorly and forms the ventral margin of the orbit (Fig 4A and 4B). It is almost twice as long as the lacrimal (in lateral view; not counting the internal anteriorly elongated process of the lacrimal) and is positioned dorsal to the attenuate posterior process of the maxilla on the zygoma. Internally, the jugal bears a flat anterior process which underlies the maxilla. This process is rectangular in shape and extends further anterior than the equivalent process in *Suchogorgon* [15]. A small horizontal crest (= lamina ventralis of Ivakhnenko [15]) is situated at the ventralmost margin of this process, reaching over half the length of the complete bone. The curved, plate-like internal orbital contribution of the jugal bears a short, blunt-ended process extending ventromedially, overlying the ectopterygoid and extending towards the pterygoid. This structure was also described as present in the gorgonopsians *Arctops* and *Suchogorgon* by Laurin [14] and Ivakhnenko [15] (respectively; although note that the specimen of *Arctops* described by Laurin was identified as *Lycaenops* in that paper [13]). The lower orbital margin also bears a horizontal ridge (= crista infraorbitalis of Ivakhnenko [15]) medially, which is thicker in MB.R.999 than that described for *Suchogorgon*. The posterior end of the jugal is missing on both sides of the skull in MB.R.999. A partial right jugal is also present but more poorly preserved than the left; it, too, only represents the anteriormost portion.

**Fig 10:**
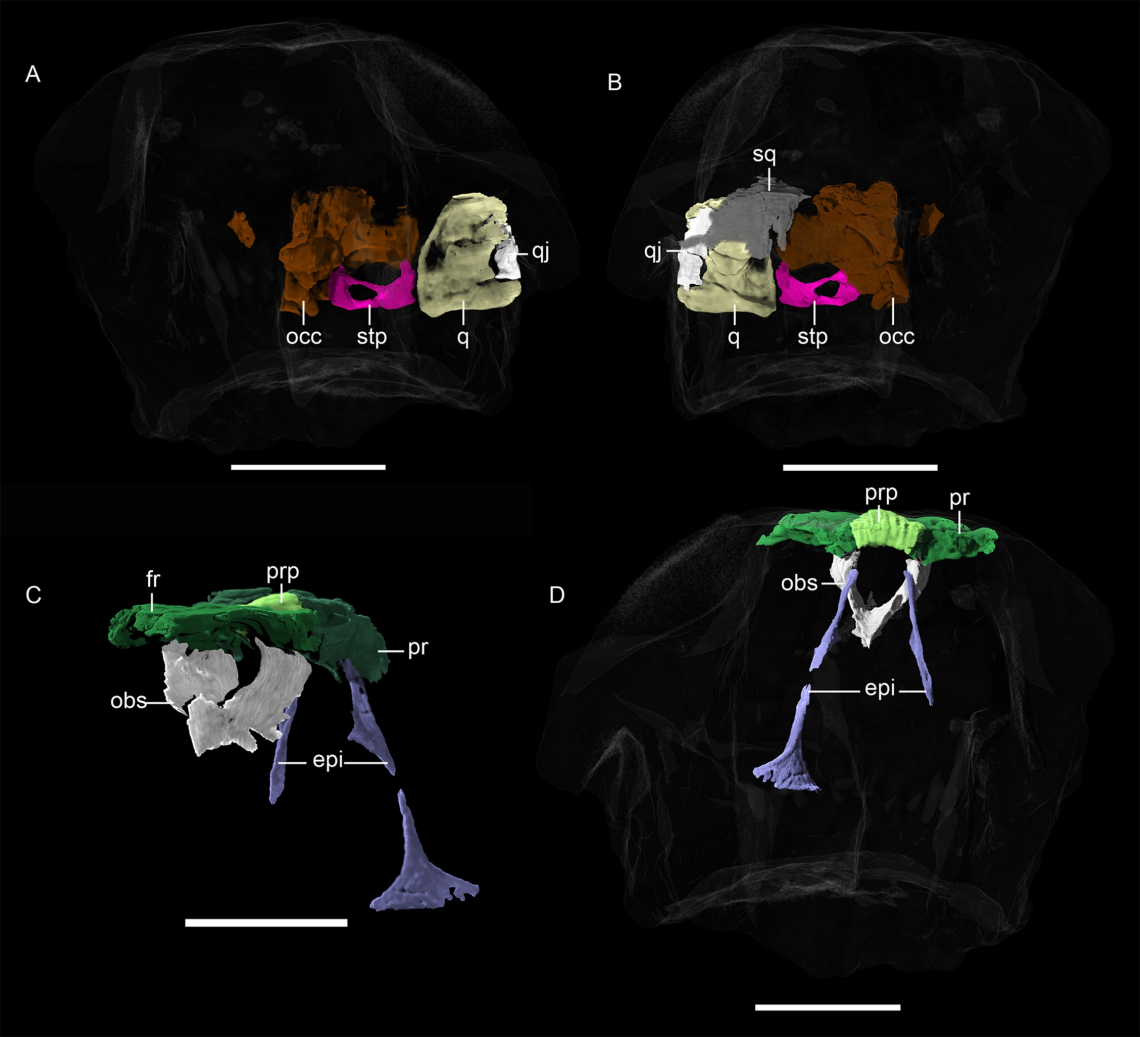
**Neutron CT reconstruction of MB.R.999: occipital region from (A) anterior and (B) posterior; epipterygoid/orbitosphenoid from (C) anterolateral and (D) posterior.** Abbreviations: epi, epipterygoid; fr, frontal; obs, orbitosphenoid; occ, fused occipital components; pr, parietal; prp, preparietal; q, quadrate; qj, quadratojugal; sq, squamosal; stp, stapes. Scale bars = 2 cm.

*Squamosal*. *—* The squamosal usually makes up the subtemporal zygoma and the posterior edge of the temporal fenestra [35]. It is, however, only preserved as a small laminar fragment in MB.R.999 (Fig 10B). All that can be said is that it covers the dorsal part of the quadrate-quadratojugal-complex to some extent and contacts the occipital plate laterally, forming a recess into which the paroccipital process of the opisthotic fits.

*Quadrate-quadratojugal complex*. *—* This structure is not preserved completely due to damage to the posterior side of the skull; moreover, only the left complex can be assessed in MB.R.999 (Figs 10A and 4B). The quadrate is an anteroposteriorly flattened structure, with a bulbous dorsal process that sits loosely (i.e. it is not sutured) in the anterior recess of the squamosal. The body of the quadrate is posteriorly convex and ventrally constricted anteroposteriorly. At its ventral extent, a saddle-shaped condyle with two slightly ventrally-protruding projections secures the articulation of the quadrate with the articular. The lateral of the two condyles is more robust than the medial, mirroring the condition described by Kemp [12]. The condyles of MB.R.999 are more confluent than described by Kemp for Tanzanian rubidgeine gorgonopsians, however. Mostly because the lateral condyle is not globular but the overall curvature is instead more subdued in MB.R.999. On the lateral side of the quadrate, it is partially covered by the laminar quadratojugal, which is poorly preserved and therefore only allows for an incomplete description: it is a ribbon-like structure reaching from the dorsal end of the quadrate to its lateral condyle. In posterior view, the quadrate-quadratojugal complex is roughly triangular in shape but with rounded corners. A large quadrate foramen is present between the two elements of the complex.

*Ectopterygoid*. *—* The ectopterygoid is a ‘saddle’-shaped palatal element situated between the palatine, maxilla, and pterygoid (Fig 5A and 5B). Its ventral surface is mostly concave, but laterally bears a well-developed ridge extending anteriorly from the tip of the transverse process of the pterygoid. Posteriorly, the ectopterygoid forms part of the anterior wall of the transverse process. No close contact between the lateral margin of the ectopterygoid and the medial border of the maxilla can be observed in this specimen, probably due to matrix filling the sutural space, but a weak suture with the jugal is evident. A weak sutural contact between this element and its adjoining bones was also described by Ivakhnenko for *Suchogorgon* [15].

**Fig 11:**
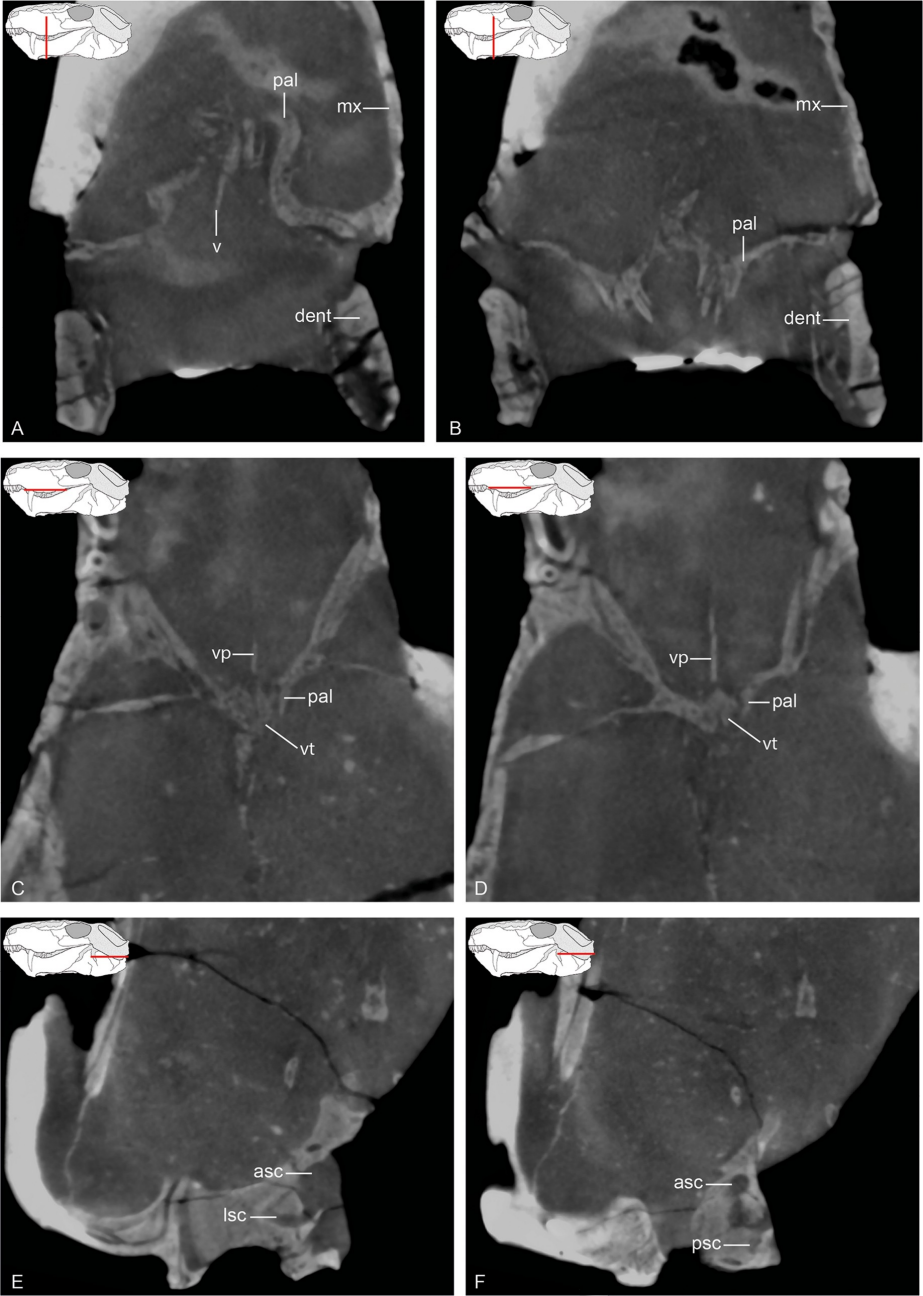
Tomographic slices of the neutron scan of MB.R.999. (A) and (B) coronal sections of the palatal region; (C) and (D) horizontal sections of suture of vomer and palatine; (E) and (F) horizontal sections of otic labyrinth region. Abbreviations: asc, anterior semicircular canal; dent, dentary; lsc, lateral semicircular canal; mx, maxilla; pal, palatine; psc, posterior semicircular canal; v, vomer; vp, posterior blade of vomer; vt; triangular plate of vomer.

*Palatine*. *—* The palatine is a dorsoventrally narrow element making up a large portion of the palatal area (Fig 5A and 5B). In lateral view, the palatine is visible in the CT scans as a wavy structure (Figs 4C, 11A and 11B). A slender, transverse ridge is located on the dorsal surface of the palatine, which was figured in Ivakhnenko’s description of *Suchogorgon* [15] (Fig 25) but not mentioned further in the descriptive text. This ridge is part of the ala maxillaris, the wing-like, anterior palatine structure that forms the contact with the maxilla via a V-shaped suture (extending dorsally) on the medial surface of the maxilla. An anteromedial indentation of the palatine surrounds the posterior tip of the vomer. Posteriorly, a triangular notch encloses the anterior tip of the fused pterygoid.

Ventrally, each palatine bears a reniform palatine boss, situated posteroventrally and bearing several small (~0.75 cm long including roots) teeth (Fig 6A and 6B). Seven conical teeth are arranged in one V-shaped row (with the apex pointed anteriorly, and the medial arm being shorter than the lateral arm) per boss. These teeth have shallow roots and are slightly posteriorly angled. Two (on the left side) and five (on the right one) very small teeth outside of the main row on each boss are probably remnants of replacement teeth.

*Vomer*. *—* The anatomy of the fused vomer is distinctive for gorgonopsians [36]. In MB.R.999, it is roughly rhombic in its dorsal outline, and anteriorly is broad and dorsoventrally flattened (Figs 4C, 5A, 5B and 5C). Posteriorly, it consists of a thin tapering blade that terminates as a small, flat triangular structure between the two palatine wings. The exact suture with the palatine is not clearly visible and its posterodorsal extent is uncertain (Fig 11C and 11D), but the vomer is definitely separated from the pterygoid as is in other African gorgonopsians [31]. The ventral surface of the vomer bears one median and two lateral ridges, all originating from the same point at the posterior tip of the vomerine body and then dividing anteriorly (Fig 5B). The median vomerine ridge extends further ventrally and anteriorly than the two lateral ones. The main vomerine body expands gradually anteriorly, but constricts slightly at its anterior tip where it is bounded laterally by the premaxillae. Dorsally, the anterior tip of the vomer has a trident-shaped terminus that extends between the lateral and medial portions of the vomerine process of the premaxillae (Fig 5A). Dorsally, the vomer has a narrow, blade-like ridge running along its entire midline, which would have served as the attachment site for a cartilaginous nasal septum [37].

*Pterygoid*. *—* The pterygoid is usually a paired element in therapsids, but seems to be fused in all known gorgonopsians [13]. No suture between the two sides of the pterygoid is visible in the scan of MB.R.999, and the pterygoid appears to be a single element (Fig 5A, 5B and 5D). The pterygoid can be subdivided into three parts: a median palatal portion, laterally-projecting transverse processes, and the slender quadrate rami extending posterolaterally. The median portion of the pterygoid bears a thin median lamina projecting dorsally into the interior of the skull. The two transverse processes are strongly curved, giving a ‘wing’-like shape, and are anteroposteriorly expanded at their medial and lateral tips. In lateral view, these processes are angled approximately 65–70° relative to the long axis of the skull. Each process bears 5–6 small (~0.25 cm long including roots), conical teeth on its curved medial section, arranged in a single line. Two palatal pterygoid bosses extend from a point anterolateral to the median origin of the transverse processes and reach further anteriorly. The lateral and anterior margins of these bosses are roughly continuous with the lateral and posterior margins of the palatine bosses, but the dental fields and medial margins of the respective bosses are distinct from one other. Each boss bears a field of 8–9 small (~0.5 cm long including roots), anteriorly-angled conical teeth (including possible replacement teeth, Fig 6A and 6C).

Posteriorly, the quadrate rami of the pterygoid are closely appressed to the parabasisphenoid anteriorly but diverge posteriorly as they extend laterally to contact the quadrates. The rather horizontal base of the ramus becomes vertical and transversely very slender posteriorly. Due to the absence of part of the right ramus, only the articulation of the left quadrate ramus with the foot of the left epipterygoid is preserved, which takes the form of a plane contact surface. Because of the close contact between the quadrate rami and the parabasisphenoid, no interpterygoid vacuity is present (as in [15] and *contra* [38]).

*Parabasisphenoid*. *—* The parasphenoid and basisphenoid (or basipresphenoid of Araújo et al. [18]) are fused, as usually is the case in adult therapsids [39] and are indistinguishable in the CT scans of MB.R.999 (Figs 4C, 5A, 5B). The tall rostrum of the parabasisphenoid is enclosed in a recess between the two quadrate rami of the pterygoid. Its dorsal surface bears an anteroposteriorly-elongate depression and ventrally, it extends beyond its contact with the pterygoid as a parabasisphenoid blade. Large triangular basal tubera form the lateroposterior limitations of the parabasisphenoid ventrally. An ascending process of the parabasisphenoid (the cultriform process [14]) arises dorsally from the bulbous posterior portion of the bone and is angled anterodorsally. The base of the cultriform process bears an oval foramen on its dorsal surface, possibly the foramen for the pituitary gland that is located at the bottom of the sella turcica (Fig 5A). The cultriform process terminates without contacting any other median braincase elements (e.g. orbitosphenoid), indicating that it could have been cartilaginous at its tip and only partly ossified during ontogeny. The parabasisphenoid usually makes up the ventral border of the braincase [12].

*Postparietal*. *—* The small, roughly triangular postparietal overlies the supraoccipital and constitutes the dorsal border of the occiput, although it is damaged in this specimen (Fig 9). Anterodorsally, it forms a narrow, pointed process that extends between the parietals. In the scan, the underlying supraoccipital is partially exposed below a broken portion of postparietal. Posteriorly, a weak nuchal ridge runs along the median surface of the postparietal from the dorsal to the ventral edge; otherwise, this surface is flat. (The indistinct nature of this ridge is probably due to wear, as this structure is usually well-developed in gorgonopsians [2, 18].) The anterior surface of the postparietal is more complex, exhibiting one median ridge and two transverse ridges originating from the medial one, forming a subtriangular notch. These two ridges extend ventrally in an arc shape.

**Fig 12:**
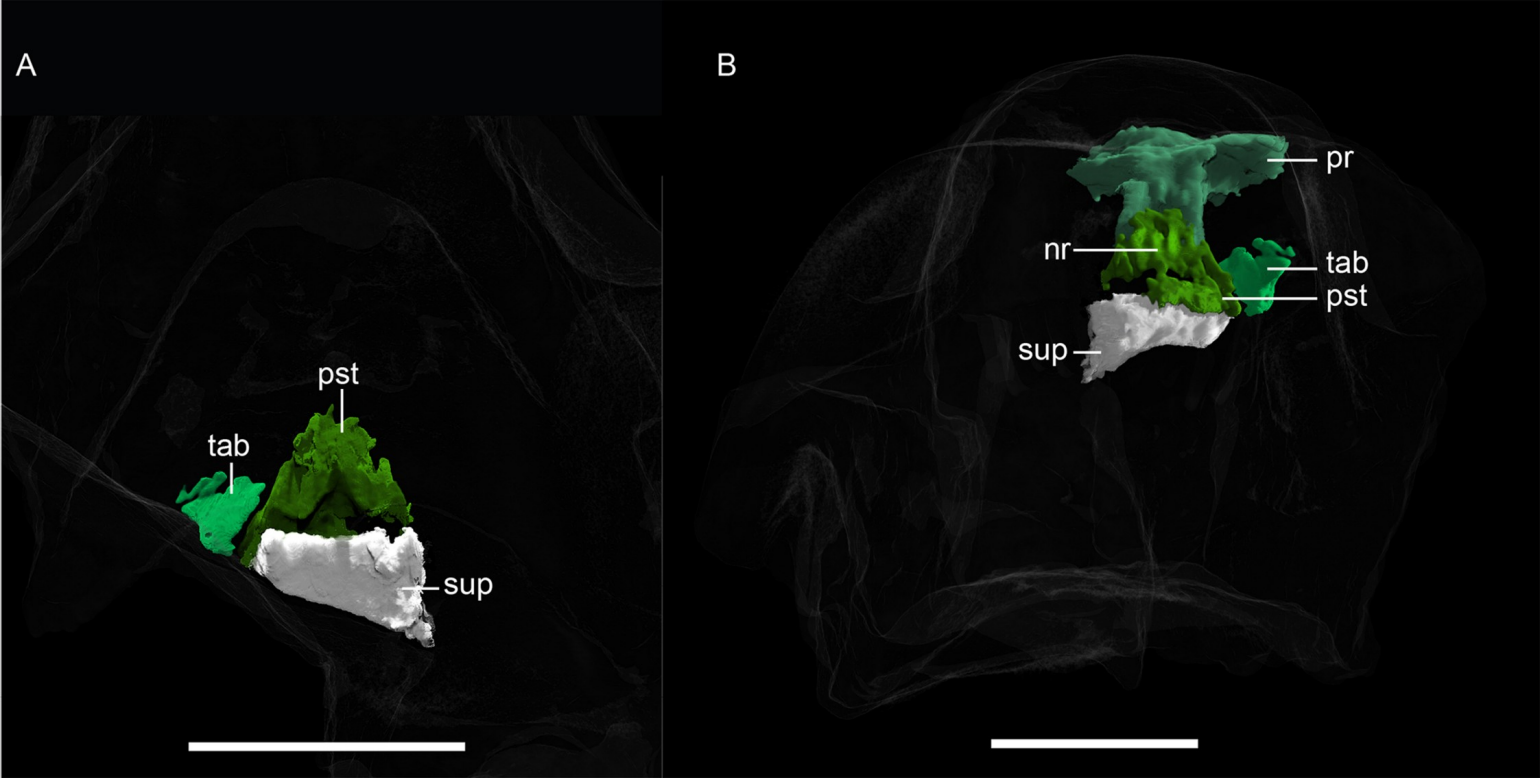
**Neutron CT reconstruction of MB.R.999: rear end of skull roof from (A) ventral and (B) dorsal.** Abbreviations: nr, nuchal ridge; pr, parietal; pst, postparietal; sup, supraoccipital; tab, tabular. Scale bars = 2 cm.

*Supraoccipital*. *—* Much like the postparietal, the supraoccipital is an unpaired median occipital element (Fig 12). Even more damaged than the postparietal, it is roughly quadrilateral with a dorsolateral process.

*Tabular*. *—* The tabular is preserved as just a fragmentary shard on the right side of MB.R.999 (Fig 12). It almost contacts the lateral side of the postparietal but is separated from it by matrix.

*Exoccipital*, *basioccipital*, *opisthotic*, *prootic*. *—* The remaining occipital elements appear fused and are indistinguishable from each other in the CT scans of MB.R.999 (Fig 10A and 10B). The paroccipital process of the opisthotic is robust and knob-like. The basioccipital (which is presumably formed of the true basioccipital fused with the basipostsphenoid following the work of Araújo et al. [18], although no separation of these elements can be seen in the scan) would have formed the occipital condyle and the posterior edge of the basicranium. The anteriormost extension of the brain case is enclosed by the paroccipital processes of the parabasisphenoid.

*Epipterygoid*. *—* Being very fragile, ribbon-like elements often obscured by matrix in the temporal fenestra, the epipterygoids are rarely observable in gorgonopsian specimens. However, they are well preserved in MB.R.999, especially the left one (Fig 10C and 10D). The left epipterygoid is missing a small (~0.3 cm) piece in the middle section but is otherwise nearly complete, whereas on the right side only the ventral half is preserved. The epipterygoid originates as a broad footplate ventrally, situated in a dorsal groove on the quadrate ramus of the pterygoid. Dorsally, it constricts and forms an elongate ascending process (= columella) before expanding again near its contact with the parietal. The contact between the epipterygoid and the ventral surface of the parietal is situated posterolateral to the pineal foramen.

*Orbitosphenoid*. *—* The orbitosphenoid is a median element composed of two medially-concave wings whose dorsal margins articulate with the ventral surface of the frontals (Fig 10C and 10D). They do not appear to contact the parietals, unlike what has been described for other gorgonopsians [3, 18]. They meet ventrally and form a keel that is taller anteriorly, but lacks the extreme height of that described for GPIT/RE/7124 [3, 18]. The anterior keel extends dorsally to form a very small ridge which separates the two lobes of the olfactory bulbs. This ridge is not as pronounced as in GPIT/RE/7124 and is only present in the most anterior preserved region of the orbitosphenoid. Whether it would have become even taller anteriorly and contacted the frontals, as in GPIT/RE/7124, is unknown, as the anterior edge is not preserved.

**Fig 13:**
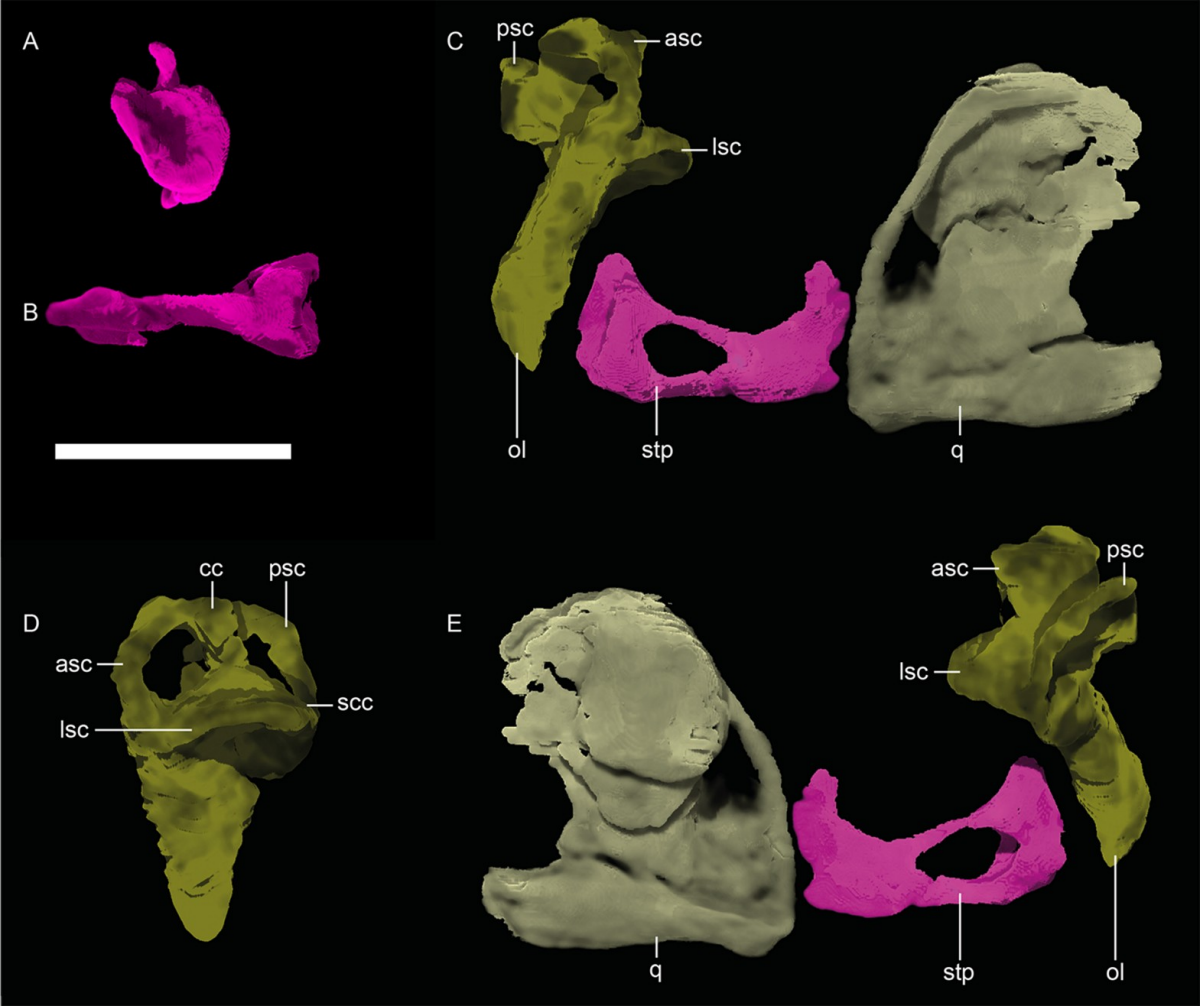
**Neutron CT reconstruction of MB.R.999: stapes from (A) medial and (B) ventral; otic ear labyrinth from (C) anterior, (D) left lateral and (E) posterior.** Abbreviations: asc, anterior semicircular canal; cc, common crus; lsc, lateral semicircular canal; ol, otic labyrinth; psc, posterior semicircular canal; q, quadrate; scc, secondary common crus; stp, stapes. Scale bar = 1 cm.

*Stapes*. *—* Only a few gorgonopsian stapes have been described, as they are often not preserved or prepared. Exceptions include those of *Sycosaurus nowaki* (UMZC T878 [2]; described as “*Arctognathus* sp.” by Kemp [12]), “*Scylacops capensis*” (UMZC T885 [38]), *Arctops willistoni* (UCMP 4270 [13]; described as “*Lycaenops angusticeps*” by Laurin [14]), and various specimens of *Suchogorgon golubevi* [15]. Thanks to the application of CT technology and the pristine condition of this delicate structure on the left side of MB.R.999, the stapes of this specimen can be described in three dimensions (Fig 13A–13C and 13E). The stapes has a medial foot plate, which is slightly concave proximally and fits into the fenestra ovalis. A dorsal plate-like process is situated at the distal side of the stapes, which together with another flat protuberance provides two contact areas for the quadrate, *contra* Kemp [12, p.76], who stated that the “*stapes probably does not abut directly on to medial face of the quadrate*.” This area differs from that of *Suchogorgon* as the ventral protuberance is larger compared to the small dorsal one in the Russian specimens where they furthermore do not display two clearly distinguishable processes but rather constitute one even distal surface. However, the short auditory ossicle bears a relatively large stapedial foramen (~30% of whole length, Table 2) in MB.R.999 as well as *Suchogorgon* with a thin dorsal margin and a more plate-like ventral one.

**Table 2:** Measurements of the stapes in MB.R.999.

*Mediolateral length*	*12*.*0 mm*
*Largest diameter of footplate*	*6*.*8 mm*
*Maximum length of stapedial foramen*	*3*.*6 mm*

*Otic labyrinth*. *—* Due to the endocranial position of the bony labyrinth of the ear, this structure is not visible in intact gorgonopsian skulls and historically was described only in sectioned or weathered specimens [40]. The few historical studies on the inner ear morphology of therapsids (e.g. [41, 42]) have recently been substantially augmented by data from CT-scanning, however [18, 40, 43–47]. MB.R.999 provides additional data on the morphology of the labyrinth in Gorgonopsia. In MB.R.999, this structure is well encapsulated but not ossified. Like the rest of the occipital region, only the left side is preserved (Fig 13C–13E). The vertical axis through the vestibule is slightly tilted posterior. The vestibule itself is roughly subtriangular in cross-section and appears longer in relation to height of semicircular canals (Table 3). No cochlear recess can be identified (in accordance with that described by Sigogneau [42]). All the semicircular canals are marginally wider than high and very alike in size. As is typical [18, 40, 44, 46], the posterior and anterior canals have their origin in the common crus. A small secondary common crus (Fig 9A) is formed at the contact of the posterior semicircular canal and the vestibule. The cross-section of the anterior and posterior semicircular canals is ovoid to circular, and the cross-sectional lumen diameter is 1.25 mm and 1.9 mm at their broadest points, respectively. No real statement can be made about these parameters for the lateral semicircular canal as it is incompletely preserved. The overall shape and relative size of the otic labyrinth is concordant with that of GPIT/RE/7124 [18], including the posterior semicircular canal forming the smallest curve.

**Table 3:** Measurements of the otic labyrinth in MB.R.999.

*Largest diameter of anterior semicircular canal*:	*3*.*4 mm*
*Semicircular canal complex height*:	*9*.*2 mm*
*Maximum length of otic labyrinth*:	*19*.*3 mm*
*Length of vestibule*:	*9*.*8 mm*
*Maximum width of vestibule*:	*6*.*0 mm*

*Brain endocast*. *—* Due to the occipital region only being partially preserved and general poor ossification of the braincase in gorgonopsians, the brain endocast could only partially be segmented (see Fig 9). In particular, the ventral limits of the brain endocast as reconstructed are mostly speculative. Nevertheless, some important features can be confidently discerned, such as the morphology and anterior extent of the olfactory bulbs. They are anteriorly and ventrally delimited by anteroposteriorly-running ridges on both frontals (see Fig 8B), which merge anteriorly. The shape of the olfactory bulbs is more globular than in GPIT/RE/7124 (the only other gorgonopsian, whose brain endocast has been described by 3D data [18]), but the midline ridge dividing the two elements is less clearly visible. The well-preserved orbitosphenoid gives one of few indications of the shape and size of the ventral limitation of the brain endocast, including the olfactory tracts, of MB.R.999, which again appears to be relatively larger than in GPIT/RE/7124. The length of the olfactory bulbs is ~ 17 mm, the maximum width is ~ 15 mm. No clear distinction can be found between the olfactory region and the forebrain.

The epiphyseal nerve, which pierces through the round pineal foramen and emerges from the forebrain, is almost half the size in diameter (~3 mm) compared to that in GPIT/RE/7124 (> 5mm). Further posteriorly the lateral margins of the endocast are encased by the epipterygoids. Here, the brain endocast reaches ventrally until the hypophysis contacts the sella turcica (Fig 5A) of the parabasisphenoid. Since the epipterygoid reaches far laterally (Fig 10C and 10D), the lateral demarcations are not well defined in this most ventral part of the brain endocast.

Posteriorly and dorsally, the short and broad hindbrain connects to the supraoccipital, parabasisphenoid and the occipital complex. The description of the hindbrain is limited in MB.R.999 due this region being damaged, but the left paraflocculus can still be located since it is partially enclosed by the anterior canal of the otic labyrinth. Its diameter is fairly slim in contrast to the one estimated for *Gorgonops* [42], but the lateral extent of this structure is not evident.

#### Mandible

The mandible of MB.R.999 is in overall good condition with its left side being almost complete, whereas the posterior part of the right side is incomplete (due to the missing portion, which was however previously briefly described by Janensch [20]).

**Fig 14:**
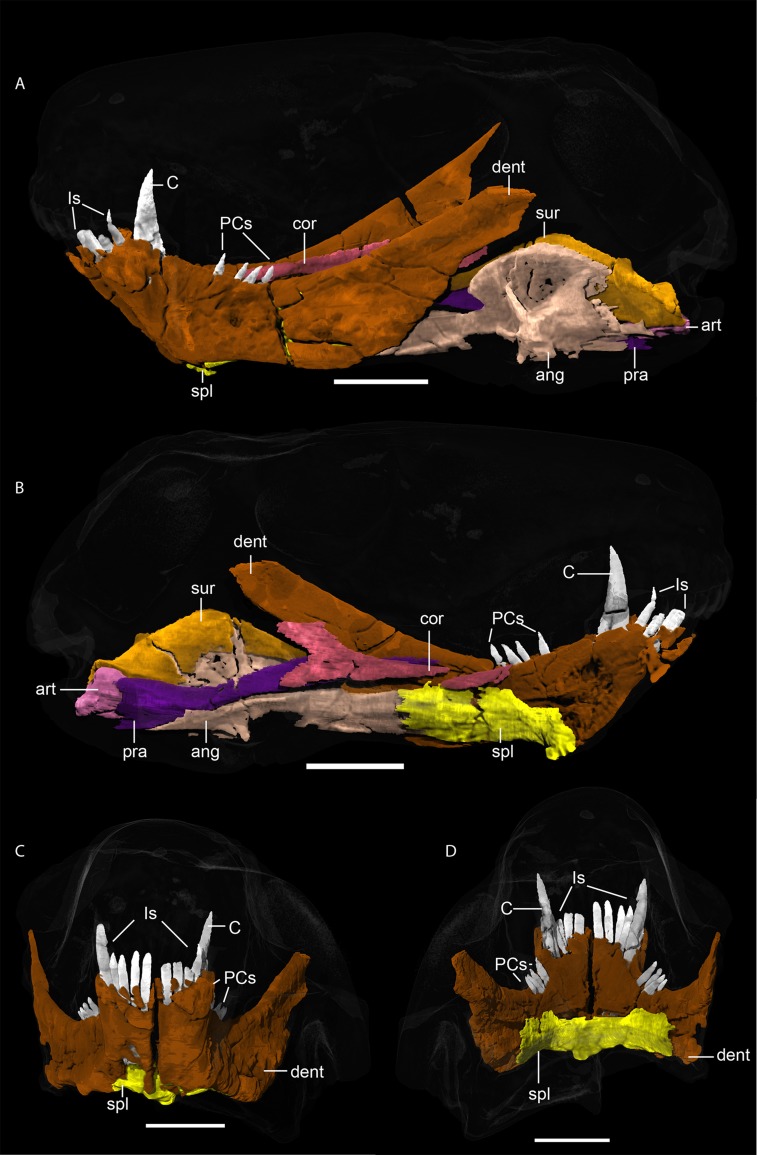
**Neutron CT reconstruction of MB.R.999: mandible from (A) left lateral, teeth on the right side digitally removed and (B) medially clipped in half; dentary and splenial from (C) anterior and (D) posterior.** Abbreviations: ang, angular; art, articular; C, canine; cor, coronoid; dent, dentary; Is, incisors; PCs, postcanines; pra, prearticular; spl, splenial; sur, surangular. Scale bars = 2 cm.

*Dentary*. *—* The dentaries (Fig 14) are robust but unfused in the symphyseal region and have slightly shifted against each other post mortem. The mandibular ramus is markedly dorsoventrally lower than the symphysis. Posterodorsally, the dentary terminates in a freestanding coronoid process, which is the transversely thinnest section of this bone. The dentary bears four conical incisors atop the symphysis, which are circular in cross-section and mostly broken at tip, but with roots reaching very deep into the alveoli (~1.25 cm). Serrations cannot be seen on the lower incisors, but are presumed to have been present based on the condition in the uppers and more generally in Gorgonopsia [4]. The single large canine (~2 cm in height, excluding the root) is ovoid in cross-section (Fig 6) and has serrations on its distal edges similar to those on the upper canine. The lower canine root is as long as the exposed crown. Four postcanines (with one replacement tooth on the right and three on the left side) are present posteriorly. They are posteriorly canted, unlike the incisors which are somewhat procumbent. Very poorly preserved, but present, serrations are located on the distal edges of the postcanines.

*Splenial*. *—* Medial to the dentary, the splenial is present as a slender, laminar element about half the length of the mandible (Fig 14). At the base of the symphysis, the splenials unite in a thickened splenial process that is typical for most gorgonopsians [2]. A distinct suture between the splenials cannot be discerned, though it is uncertain whether they were truly fused or whether the suture is just poorly preserved.

*Angular*. *—* The angular extends between the dentary and splenial anteriorly and covers most of the postdentary bones laterally at its posterior end (Fig 14A and 14B). The dominant feature on its lateral surface is the large, plate-like reflected lamina showing the characteristic ornamentation for Gorgonopsia: cruciate, with one strongly-developed, dorsoventrally-oriented bar and a less distinct anteroposteriorly-oriented one. This lamina terminates well anterior to the jaw articulation. Medially, the anterior end of the angular (between the dentary and splenial) consists of two prongs, the ventral one larger than its dorsal counterpart, which then merge into a distinct ridge that extends posteriorly. The posterior medial surface of the angular is divided at mid-height by a groove housing the prearticular.

*Surangular*. *—* Only the left surangular is preserved in MB.R.999 (Fig 14A and 14B), forming a large part of the posteriormost part of the mandible dorsal to the angular. Anteriorly it inserts between the prearticular and dentary as a narrow lamina. The dorsal margin of the surangular is thickened, forming a ridge. A lateral concavity is present under this ridge at its posteriormost edge. Posteriorly, above where the surangular has a suture with the articular, it curves dorsally, forming a short process.

*Coronoid*. *—* The coronoid is typically a small, triangular bone in gorgonopsians [3], although this could be due to incomplete preservation. In MB.R.999, however, it is an elongate and laminar bone (Fig 14), especially anteriorly, not unlike that of *Leontosaurus vanderhorsti* [2]. A thin segment of bone anteriorly, which extends beneath the fourth postcanine (Fig 14B), is here identified as a broken anterior portion of the coronoid. It could also be part of the prearticular, although appears disconnected from that bone by matrix, and occupies a more medial position. The posterior end of the coronoid is chevron-shaped, with a posterodorsally-directed ascending process. In contrast to the specimen of *Sycosaurus* described by Kemp ([12]; “*Arctognathus* sp.” therein), this part is laminar, like the rest of the bone.

*Prearticular*. *—* The prearticular is a thin, elongate bone extending from near the posterior tip of the jaw (where it contacts the articular) through a medial groove on the angular and terminating up against the medial face of the dentary (beneath the coronoid process) (Fig 14). The posterior end of the prearticular is ‘shovel’-shaped, concave laterally and forming a small process medially. The concave face accommodates the front end of the articular.

*Articular*. *—* The robust articular is a roughly pyramid-shaped bone (Fig 14A and 14B) as preserved. However, due to damage to the medial side of the bone, its complete morphology is not known; for example, the extent of the retroarticular process is uncertain (this structure usually forms a well-developed ‘hook’ in gorgonopsians [2, 12]). A slight posterior depression provides the articulation surface for the quadrate and another, although smaller, groove can be identified on the ventral surface.

## Discussion

### Taxonomic identity of MB.R.999

There remains considerable uncertainty surrounding the alpha taxonomy of small-bodied gorgonopsians. Gorgonopsian taxonomy in general has long been problematic; following the superfluity of taxa named in the first half of the 20th Century, there was a 50-year period in which only the monographs of Sigogneau [3, 4] seriously addressed the issue. Recently, more extensive revisionary work on gorgonopsian taxonomy has been published [2, 13, 16, 17, 32, 35, 48], but with some exceptions [16, 17, 32] these papers have mostly addressed the large-bodied taxa. Small gorgonopsians are especially troublesome from a taxonomic standpoint because the possibility that they represent juveniles of taxa with large adult body size is difficult to discount, particularly given their often-poorly-preserved and prepared type material. CT-scanning can help ameliorate this issue, however, both by permitting a better understanding of the morphology of historic holotypes and by providing information on the degree of cranial fusion in small gorgonopsian skulls. Although degree of sutural closure is not a definite proxy for maturity in amniotes (see, e.g. [49, 50]), fusion of cranial elements typically does characterize adult individuals of modern synapsids [51–54].

MB.R.999 shows the maximal degree of cranial fusion known in gorgonopsians, comparable to that seen in the largest known individuals of large-bodied taxa [2, 35]. The basioccipital (presumably including the basipostsphenoid), exoccipitals, and opisthotic appear to form a single fused element, and the parabasisphenoid (parasphenoid+basipresphenoid of [18]) is fully fused. These elements were still unfused in a specimen (GPIT/RE/7124) of similar length to MB.R.999 that Araújo et al. [18] interpreted as juvenile gorgonopsian. As such, we interpret MB.R.999 as an adult individual of a small-bodied taxon, rather than a juvenile.

Historically, two generic identifications have been given for MB.R.999. The specimen was labeled in the Museum für Naturkunde collections catalogue as *Aelurognathus* and briefly described by Janensch [20] as *Aloposaurus*? sp. This specimen is clearly not referable to *Aelurognathus*, which is a much larger (~30 cm basal skull length) gorgonopsian with an anteroposteriorly-expanded postorbital bar (evident even in smaller specimens interpreted as juveniles) and posteriorly-expanded palatal premaxillary body [2]. Comparisons with *Aloposaurus* are more difficult. A variety of specimens have been referred to this genus [4], many of them apparently immature [3] and of highly uncertain congenericity. The type species of *Aloposaurus*, *A*. *gracilis*, is based on a small (10.0 cm basal skull length), badly-crushed skull and lower jaws (AMNH FARB 5317) in which the cranial sutures and palatal morphology are hard to discern. An assessment of its validity is beyond the scope of the current paper, but we can note that compared with MB.R.999, this specimen has a proportionally longer, lower snout, shorter upper postcanine tooth row, and a preparietal that originates at the anterior edge of the pineal foramen (which is situated on a boss). Given the fact that proportional snout length generally increases through ontogeny rather than the reverse (including in gorgonopsians with known growth series [35]), we consider the differences between these specimens to reflect taxonomic rather than ontogenetic variation, and do not support referral of MB.R.999 to *Aloposaurus*.

Of the small-bodied gorgonopsian genera recognized by Sigogneau [3, 4], some (e.g. *Cerdorhinus*, *Paragalerhinus*) are known from limited, extremely poor material that in the absence of further preparation or CT-reconstruction must be considered nomina dubia. However, three of her small-bodied gorgonopsian genera (*Aelurosaurus*, *Cyonosaurus*, and *Scylacognathus*) are represented by extensive material, at least some of which is well preserved and fully prepared, permitting comparison with MB.R.999. Of these three, *Cyonosaurus* is most clearly distinct from MB.R.999—like *Aloposaurus*, *Cyonosaurus* has a proportionally much longer, lower snout than MB.R.999. It also has a distinctive postcanine row consisting of 6–7 extremely small teeth in a close-packed section occupying the center of the maxilla, widely separated from the canine. Finally, *Cyonosaurus* typically has a posterolateral ‘bend’ to the postfrontal at its posterior edge, giving it a more ‘wing’-shaped morphology than is present in MB.R.999.

*Aelurosaurus* is superficially similar to MB.R.999, and indeed was considered to be a possible identification for this specimen earlier in the research history of this project [47]. A number of species have been referred to *Aelurosaurus* [3], all of which are represented by small (estimated basal skull lengths 8–15 cm) skulls with proportionally short snouts and 5–6 upper postcanines (similar to MB.R.999). The validity of the various referred species is questionable and requires additional attention, but the type species, *A*. *felinus*, does appear to represent a distinct, small-bodied gorgonopsian taxon, with a palatal morphology similar to *Gorgonops* (extensive dentition on the palatal bosses and transverse processes of the pterygoid, delta-shaped palatine bosses [16]) but a shorter, taller snout and higher tooth count. Despite their general cranial similarity, several important palatal characters distinguish *A*. *felinus* from MB.R.999: in *A*. *felinus* (best represented by the acid-prepared holotype, NHMUK PV R339), the palatine bosses are delta-shaped, whereas they are reniform in MB.R.999; the palatal boss of the pterygoid is proportionally larger and more densely dentigerous in *A*. *felinus* than MB.R.999; and the vomerine body of *A*. *felinus* is the same transverse width through most of its length, which is also equivalent to the width of the vomerine process of the premaxilla. In MB.R.999, by contrast, the vomerine body continuously expands anteriorly until it is surrounded by the yet-wider vomerine process of the premaxilla. The blade-like posterior portion of the vomer is also proportionally longer in MB.R.999 than in *A*. *felinus*.

The genus *Scylacognathus* was recently revised in part by Kammerer et al. [17], who considered its type species (*S*. *parvus* from the *Tapinocephalus* AZ) to be synonymous with *Eriphostoma microdon* (which they considered the only valid species of middle Permian gorgonopsian in South Africa). However, they left the status of the two remaining species of *Scylacognathus* (*S*. *grimbeeki* and *S*. *robustus*) as uncertain pending future work. *Scylacognathus grimbeeki* was originally described by Broom [55] as *Cynarioides grimbeeki*, and was named on the basis of a crushed, incompletely prepared skull and lower jaws (TM 245) from Leeuwpoort, near Beaufort West (*Tropidostoma* AZ). Sigogneau [3, 4] considered a second nominal species of *Cynarioides* (*C*. *laticeps* [55]), represented by another crushed skull and jaws (TM 246) from Kookfontein, near Victoria West, to be synonymous with *S*. *grimbeeki*. *Scylacognathus robustus* was originally described by Broom [56] as *Cynariops robustus*, and was named on the basis of a partial skull (missing the temporal arches) (NHMUK PV R5743) from Biesiespoort, near Victoria West (the same locality where MB.R.999 was collected).

The stratigraphy of therapsid-bearing rocks near Victoria West, where several of the aforementioned small gorgonopsians have been found, is problematic, as they exhibit an apparent conflict in local bio- and lithostratigraphy. Broom [56, 57] considered these exposures to represent ‘*Endothiodon* Zone’ (= *Tropidostoma* AZ) and lower *Cistecephalus* Zone strata, a conclusion supported by subsequent collecting in the area [58]. However, geological mapping of the area has indicated that the majority of these exposures pertain to the Poortjie Member of the Teekloof Formation, which corresponds to the earlier (middle or earliest late Permian) *Pristerognathus* AZ elsewhere in the basin [59]. More recent collecting and mapping of the area suggests that there is diachroneity in the appearance of the Poortjie, and that these exposures do indeed represent the *Tropidostoma* and *Cistecephalus* AZs [60]. Regarding the gorgonopsian specimens in question, NHMUK PV R5743 and MB.R.999 both come from the upper *Tropidostoma* AZ and TM 246 comes from a probable zone of overlap between the *Tropidostoma* and *Cistecephalus* AZs [60].

**Fig 15:**
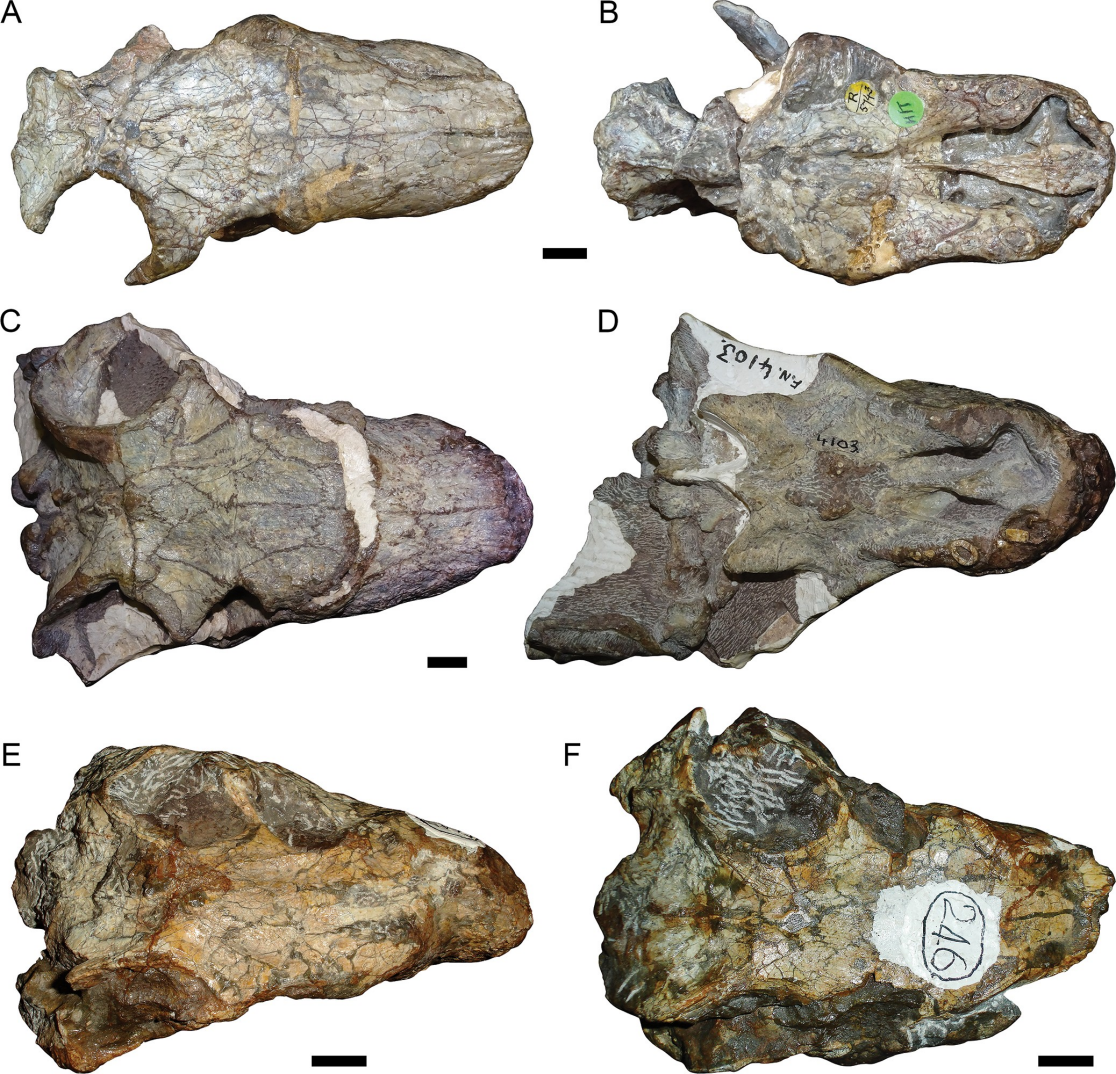
Type and additional referred material of *Cynariops robustus* Broom 1925 [56]. (A) NHMUK PV R5743, holotype of *Cynariops robustus*, in dorsal and (B) ventral views. (C) BP/1/4103, referred specimen of *C*. *robustus*, in dorsal and (D) ventral views. (E) TM 245, holotype of *Cynarioides grimbeeki*, in dorsal view. (F) TM 246, holotype of *Cynarioides laticeps*, in dorsal view. Scale bars = 1 cm.

MB.R.999 accords extremely closely with the morphology of NHMUK PV R5743 (Fig 15A and 15B). Both specimens have similar skull proportions (short snout with a tall anterior edge, not low and broad as in *Gorgonops*), comparable tooth counts (five upper postcanines in MB.R.999, five right and four left upper postcanines in NHMUK PV R5743), large, ‘arrowhead’-shaped preparietals with highly interdigitated edges, and very similar palatal morphologies. In particular, the anterior palatal morphology of these two specimens is nearly identical, and otherwise unique among gorgonopsians. The combination of the main body of the vomer gradually increasing in size anteriorly (as opposed to remaining the same width like in *Aelurosaurus*, *Arctognathus*, and *Inostrancevia*), straight lateral vomerine ridges running parallel to the edge of the vomer (as opposed to forming wavy or lobate edges like in *Arctops*, *Gorgonops*, and *Sauroctonus*), transversely expanded vomerine process of the premaxilla surrounding the vomer anteriorly (as opposed to being the same width as the vomer like in *Aelurosaurus*, *Arctognathus*, and *Suchogorgon*), and anteroposteriorly short main palatal body of the premaxilla (not forming an expanded shelf posterior to the incisors, like in *Arctops* and rubidgeines) serves to diagnose a unique gorgonopsian morphotype corresponding to Sigogneau’s [3, 4] *Scylacognathus robustus*. As the genus *Scylacognathus* is now a synonym of *Eriphostoma* (based on synonymy of the type species *S*. *parvus* with *E*. *microdon* [17]), however, the genus *Cynariops* should be resurrected for this species.

In addition to MB.R.999, several additional specimens can be referred to *Cynariops robustus*. Although the anterior palate is not exposed in TM 245 (Fig 15E) and TM 246 (Fig 15F) (the holotypes of *Cynarioides grimbeeki* and *C*. *laticeps*, respectively), these specimens show comparable cranial proportions to NHMUK PV R5743 and MB.R.999 and, importantly, a preparietal with highly interdigitated edges and a pointed tip situated somewhat anterior to the pineal foramen. BP/1/4103 (Fig 15C and 15D), a distorted but nearly-complete skull from Matjiesfontein, Western Cape Province, also has comparable snout proportions, 4–5 upper postcanines, reniform palatine bosses, a gradually expanding main body of the vomer with straight lateral vomerine ridges, and an ‘arrowhead’-shaped preparietal with highly interdigitated edges. This specimen also has unusually large postfrontals, similar to that described by Broom [55] for *Cynarioides laticeps*. All known specimens of *Cynariops robustus* are from the *Tropidostoma* AZ (or potentially the *Tropidostoma*-*Cistecephalus* transition zone in the case of TM 246) and interestingly, most were found in the area south of Victoria West.

### Phylogeny

*Cynariops robustus* was included in the most recent phylogenetic analysis of Gorgonopsia (that of Kammerer & Masyutin [32], a revised version of previous analyses [2, 13]). Character codings were made in Mesquite, version 3.3 (build 854) [61] and run in the program PAUP*, version 4.0 (build 162) [62], using the branch-and-bound algorithm. Nodal support was evaluated using Bootstrap analysis (with 1000 replicates). Bremer support indices were calculated using TreeRot, version 3 [63]. Two operational taxonomic units (OTUs) for *Cynariops* were initially coded: one based solely on MB.R.999 and one including data from all five known specimens of *C*. *robustus* (BP/1/4103, MB.R.999, NHMUK PV R5743, TM 245, and TM 246, see S2 Fig; and S3 Fig for sole coding of MB.R.999). The codings for these OTUs differed only in that fewer characters could be coded for MB.R.999 (i.e. there was no character conflict between specimens) and when run in separate analyses they occupied the same position on the tree. The following treescores and support values are based on the more complete composite coding for *C*. *robustus* (NEXUS file containing data matrix in S1 File).

**Fig 16:**
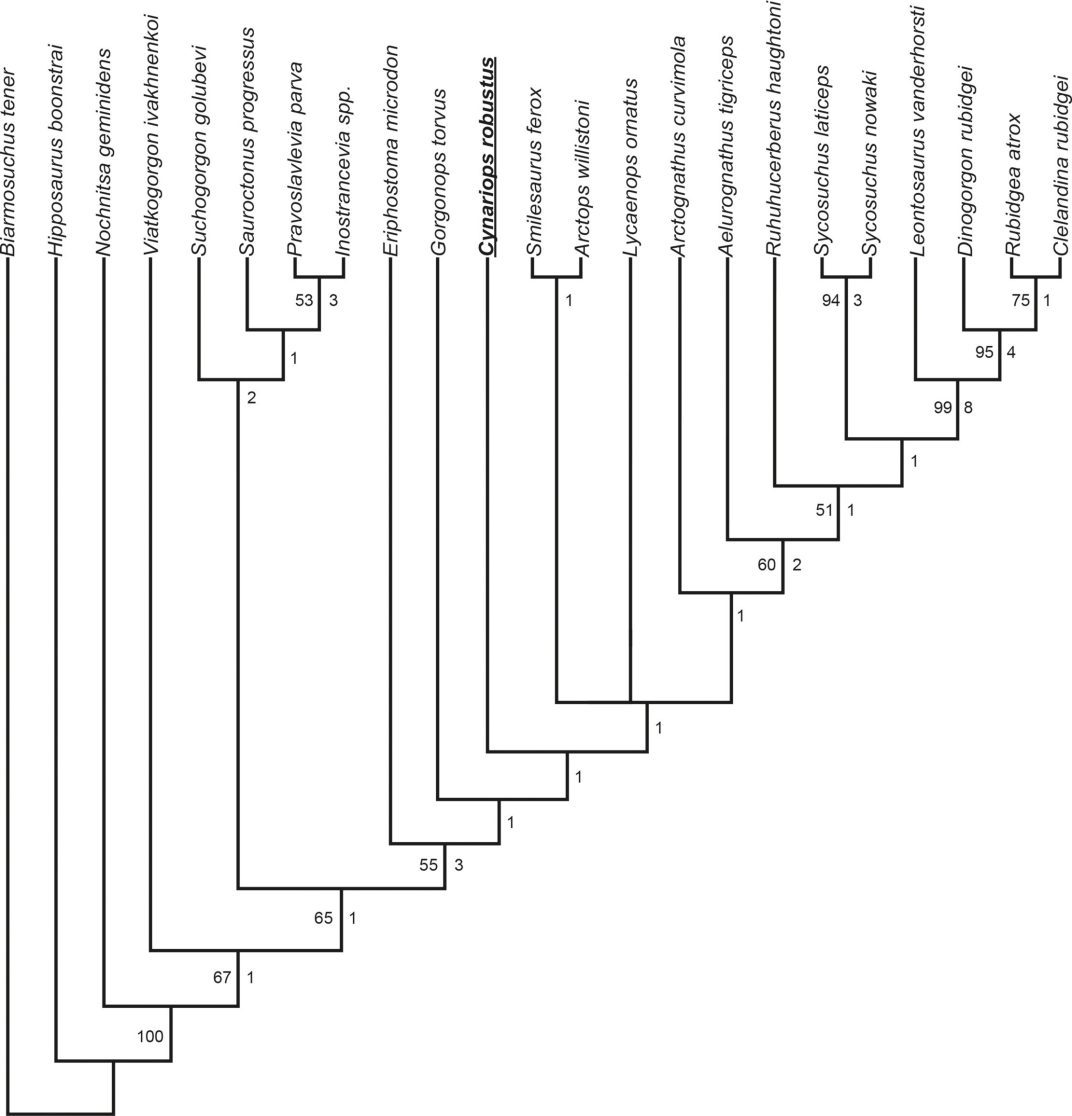
Strict consensus tree of gorgonopsian phylogenetic analysis, including MB.R.999 (= *Cynariops robustus*). Numbers left under nodes indicate bootstrapping support values above 50%. Numbers right under nodes show Bremer support indices.

The phylogenetic analysis resulted in two most parsimonious trees (score of best tree found = 102, consistency index = 0.467, retention index = 0.687, rescaled consistency index = 0.321, homoplasy index = 0.533), differing only in the positions of *Arctops willistoni*+*Smilesaurus ferox* and *Lycaenops ornatus* (as was also the case in the analysis of Kammerer & Masyutin [32], the new analysis differs only in the addition of *Cynariops*). The strict consensus tree (Fig 16) recovered *Cynariops robustus* as the sister taxon of a clade containing all African gorgonopsians other than *Gorgonops torvus* and *Eriphostoma microdon*. *Cynariops*, *Eriphostoma*, and *Gorgonops* are among the most generalized African gorgonopsians (although *Gorgonops* is somewhat unusual in its broad, flat snout) and share a large number of plesiomorphic characters (in total, these taxa share 40 out of a total of 52 character states). The slightly more deeply-nested position of *Cynariops* is supported by the presence of a reniform (rather than delta-shaped) palatal boss, deflection of the subtemporal zygoma, and proportionally shorter parasphenoid rostrum.

### Morphological variation in small-bodied gorgonopsians

Although historically poorly studied, there are a few recent papers describing small-bodied gorgonopsian specimens in some detail [15, 17, 18, 47]. With the addition of *Cynariops* to this roster, it is now beginning to become possible to assess morphological variation within this gorgonopsian size class, beyond noting simple proportional differences and general gracility relative to larger-bodied forms. Gorgonopsians are usually considered rather conservative in their morphological traits, especially compared with the other major therapsid clades [17, 35], and our current research does not overturn this assertion. However, within the basic framework of the gorgonopsian skull some areas of considerable variability are present, as we will detail below.

The vomer and vomerine process of the premaxilla have proven to be highly variable in gorgonopsians and of substantial taxonomic utility. Recent research has identified autapomorphic vomerine morphologies in a number of gorgonopsian taxa (e.g. *Arctognathus*, *Arctops*, *Gorgonops*, *Sycosaurus* [2, 13, 35] a list to which *Cynariops* may be added. In particular, the vomerine morphology of *Cynariops* is important in distinguishing it from the otherwise-similar small-bodied genus *Aelurosaurus*. Degree of vomerine expansion throughout its length and the relative positions, degree of splay, and shape of the three vomerine ridges appears to be species-specific in many gorgonopsians, and should be noted whenever possible in future descriptive work on the group. Although often hidden under matrix in historic material, digital imaging methods now allow these delicate structures to be visualized without fear of damage to the specimen [64] (unfortunately, many historical specimens were also overprepared, frequently destroying the vomerine ridges in cases where the vomer was exposed). Variation in vomerine morphology has also proven useful at higher taxonomic levels for separating Russian gorgonopsians from African ones, with the former group retaining a contact between the vomer and pterygoid that is lost in the latter [32].

The morphology of the preparietal was often emphasized as a feature of taxonomic utility in early gorgonopsian descriptions (e.g. [55–57]). Although subsequent study has questioned the degree of utility for this feature, noting intraspecific variability in exact shape (e.g. [17]), it is nonetheless clear that some differences in morphology in this element do reflect taxonomic distinction (e.g. [13]). The referred material of *Cynariops robustus* shows a generally-consistent preparietal morphotype, although the shape of this element does vary slightly between specimens (with a more pointed anterior tip in BP/1/4103 than TM 245, for instance; see Fig 15). Intriguingly, the CT data reveals unexpected variation in the internal surface of this element relative to that previously known for gorgonopsians: in MB.R.999, the ventral surface of the preparietal is ornamented with longitudinal ridges, unlike the Russian *Suchogorgon* in which it is smooth [15]. The position of the preparietal on the skull also seems to have taxonomically-relevant variability: in *Suchogorgon* the preparietal immediately abuts the pineal foramen, whereas in *Cynariops* (and various other taxa, e.g. *Arctops* [13]) it is separated from it by a short expanse of parietal.

Other internal bone surface features revealed through CT imaging may also be of taxonomic importance, although it is difficult at present to assess their variability in the clade as a whole given limited available data. For example, both *Suchogorgon* and MB.R.999 exhibit an anteriorly-directed internal process of the jugal that overlaps a substantial medial portion of the maxilla (Fig 4B), but this process is notably longer in MB.R.999 than *Suchogorgon*. Both gorgonopsians also have a thickened internal rim of the orbit, which on the jugal continues ventrally as a distinct ridge, but this ridge is much thicker in MB.R.999 than in *Suchogorgon*. The morphology of the coronoid, a small, laminar bone rarely visible in gorgonopsian specimens, also clearly differs between *Suchogorgon* and MB.R.999, having a lower, wider dorsal ramus in the former taxon [15]. For this element some additional data is available on other species, and suggests that differences in coronoid shape at least characterize different gorgonopsian subclades (in the rubidgeine *Leontosaurus*, for instance, the dorsal ramus of the coronoid is a curved, attenuate structure [2]).

These examples illuminate the potential for rarely-studied, historically hard-to-see elements to expand our understanding of cranial variation and potentially taxonomically-informative characters in Gorgonopsia. It is likely that further variation in other aspects of internal morphology (e.g. of the otic labyrinth or neurovascular morphology) is present throughout the group, but cannot at present be assessed in the absence of detailed sections or CT data for a range of gorgonopsian species. Moving forward, it will be important to more broadly sample such data for Gorgonopsia, in order to appreciate their diversity not only in terms of raw size and more obvious characters like cranial pachyostosis, but also in their more subtle and hidden details.

### Tooth replacement

The synapsid fossil record encompasses the transition between taxa with continuous replacement of (usually) homodont teeth (polyphyodont dentition), like in reptiles, and the mammalian condition of only two sets of (usually) heterodont teeth (diphyodont dentition). The gorgonopsian dentition represents an intermediate stage between these two extremes: the dentition is strongly heterodont, with differentiation into distinct incisors, canines, and postcanines (homologous with the mammalian premolars + molars), but was replaced throughout the lifetime of the animal. All gorgonopsian teeth are thecodont and unicuspid and the canines are hypertrophied and blade-like. In MB.R.999, the dental formula is is $I\frac{5}{4}$ $C\frac{1}{1}$ $\mathrm{PC}\frac{5}{4}$. CT-scans of the dentition of MB.R.999 reveal a tooth replacement pattern in accordance with that shown by Kermack [34] for other gorgonopsian taxa, with alternating replacement of the upper canines and development of replacement teeth lingually for the incisors and postcanines. All teeth that we have identified as replacements in MB.R.999 are still fully enclosed in bone; none were even partially erupted at the time of death.

The left upper incisors all have one replacement tooth in place, situated lingual to the functional incisor (Fig 6F). In the third incisor position, the replacement tooth is located lingual to the remnant of an old root. In the mandible, all but the second incisor positions show replacements (roots of older incisors being resorbed are present at the second positions). On the more complete left side of the skull, both canine alveoli have a tooth in place: the posterior alveolus bears the functional canine and the anterior alveolus holds the remnant root of a previous tooth (Fig 6E). Lingual to them, replacement canines are developing, one behind the functional canine and two behind the remnant (the more medial one being the smallest). The replacement canine would move buccally to fill the alveolus after the functional tooth was shed and its root resorbed completely. A single alveolus for the lower canine is present (Fig 6D). On the right side, two replacement canines are situated lingual to the functional one. On the left, only one replacement is visible.

Both upper and lower postcanines exhibit more limited replacement than the other tooth types and are inferred to have replaced more slowly. In the left upper jaw, the root of the third postcanine is not long as the others and may have been about to fall out, but a replacement associated with this tooth position is not evident. The second postcanine shows a clear replacement lingually, however. Although difficult to discern in the scan, more replacement teeth appear to be present in the lower jaw. On the left side three tooth positions seem to bear replacements or remnants (anterior to the first and second, as well as posterior to the fourth) and on the right all four do (with both a remnant anterior and replacement posterolingual to the first postcanine).

The style of incisor replacement displayed in MB.R.999 is referred to as being "brisk" by Hopson [65], with rapid succession of the entire incisor row instead of alternating patterns of replacement between adjacent incisors. By contrast, alternating replacement of the upper canines was definitely present and is typical for gorgonopsians (and basal therocephalians), as shown by Kermack [34]. The replacement history of the upper postcanines is somewhat different, although some degree of alternation seems to have been present: the uneven-numbered tooth positions (1, 3, and 5) are slightly larger than the even-numbered ones (2, 4) in MB.R.999, with the largest tooth present in the first tooth position. Here, parallels to the tooth replacement pattern of anomodonts as described by Hopson [65] can be drawn. He suggested that some kind of alternation is definitely present, referring to the *Zahnreihen* (German for "tooth row") theory of Edmund [66]. This theory hypothesized that a certain stimulus for the production of new teeth originates mesially and moves posteriorly. However, the validity of *Zahnreihen* theory in fossil amniotes remains debatable, and has been found not to be accurate in studies of extant reptiles [67–69].

The replacement history of the various tooth types in gorgonopsians is in keeping their utility. The blade-like canines were critical for prey capture, so maintaining two functional canines throughout the replacement process was vital, and their rapid replacement essential. In addition to having canines developing in both anterior and posterior alveoli simultaneously, multiple generations of replacement teeth are visible in MB.R.999 (at least associated with the left anterior alveolus) to ensure that a canine was “waiting in the wings” at all times. Although presumably less critical for killing prey (at least in terms of needing every tooth fully erupted), gorgonopsians also probably relied heavily on the incisors for prey manipulation, making rapid replacement useful. Gorgonopsians would have used the (usually serrated) incisors to tear off chunks of prey, which then would have been swallowed whole. Postcanines seem to have been less essential, permitting their slower replacement, and indeed were reduced or lost completely in several lineages of gorgonopsians [2].

### Conclusions

The taxon *Cynariops robustus* is resurrected, and contains small-bodied African gorgonopsians characterized by an anteriorly gradually expanding vomer and large vomerine processes of the premaxilla, laterally enclosing the vomer anteriorly. Redescription of this taxon based on CT scans of an undistorted specimen (MB.R.999) reveals novel aspects of the bony anatomy of *Cynariops* as well as information on the cranial endocast and otic labyrinth. Although *Cynariops* is distinct from other small-bodied gorgonopsian taxa (e.g. *Aelurosaurus*, *Cyonosaurus*), the taxonomic status of most small-bodied gorgonopsians remains uncertain, and additional revisionary work on these taxa is necessary to provide a firm understanding of diversity in small-bodied amniote predators in the late Permian.

## Supporting information

S1 Fig Label of MB.R.999. Photocopy of the label for specimen MB.R.999 in the collections of the Museum für Naturkunde, Berlin.
(PDF)

S2 Fig Strict consensus tree of gorgonopsian phylogenetic analysis, MB.R.999 and referred specimens of *Cynariops robustus* coded as separate OTUs. Numbers left under nodes indicate bootstrapping support values above 50%. Numbers right under nodes show Bremer support indices.
(TIF)

S3 Fig Strict consensus tree of gorgonopsian phylogenetic analysis, only MB.R.999 and no other referred specimens of *Cynariops robustus* coded. Numbers left under nodes indicate bootstrapping support values above 50%. Numbers right under nodes show Bremer support indices.
(TIF)

S1 File Data matrix of this study for both MB.R.999 and a composite coding of other referred specimens of *Cynariops robustus*, available in NEXUS format. (NEX)

S2 File CC BY Permit of Carola Radke, Mfn. For reprint [22] and use of Figs 1–3 under a CC BY license, original copyright 2016
(PDF)
