# Supplementary material for: Cranial anatomy of the gorgonopsian Cynariops robustus based on CT-reconstruction
Source: PLoS One. 2018 Nov 28;13(11):e0207367. doi: 10.1371/journal.pone.0207367 (PMC6261584; doi:10.1371/journal.pone.0207367)
Supplement: S1 Fig — Photocopy of the label for specimen MB.R.999 in the collections of the Museum für Naturkunde, Berlin. (PDF) [file pone.0207367.s001.pdf]

MB. R. 999 — Aelurognathus sp.
MB.I.027.21 — Schädel mit Unterkiefer

Oberes Perm — Biesjespoort,
Bez. Beaufort West,
Kapprovinz, Südafrika

leg. W. JANENSCH 1929
Alte Kat.-Nr.: I.Nr.14.1929

Bemerkungen: Expl. befindet sich in der Ausstellung (Sauriersaal) ✓
Saal II
