# Supplementary figures and images for: Cranial anatomy of the gorgonopsian Cynariops robustus based on CT-reconstruction
Source: PLoS One. 2018 Nov 28;13(11):e0207367. doi: 10.1371/journal.pone.0207367 (PMC6261584; doi:10.1371/journal.pone.0207367)

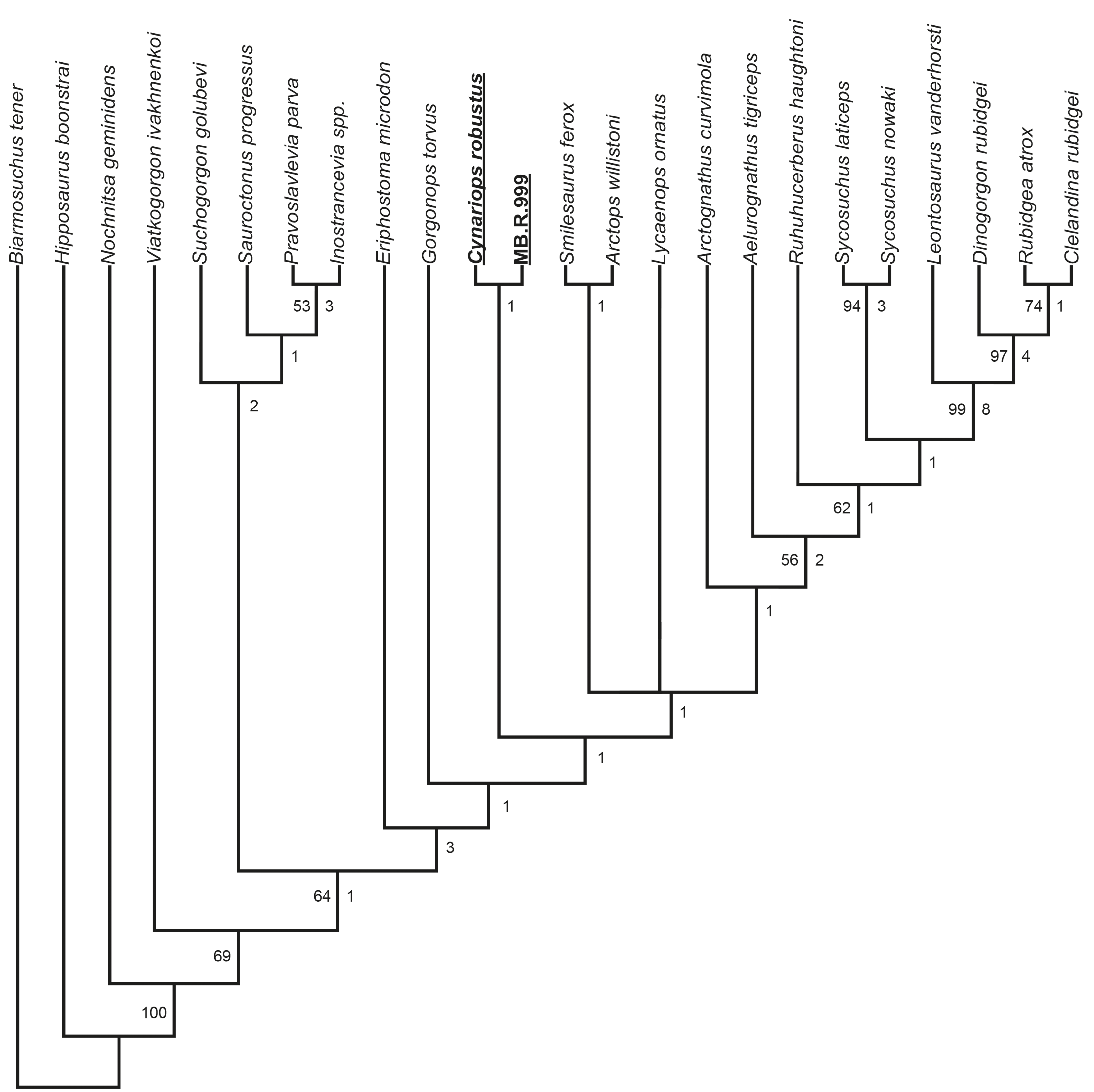

Supplement: S2 Fig — Numbers left under nodes indicate bootstrapping support values above 50%. Numbers right under nodes show Bremer support indices. (TIF) [file pone.0207367.s002.tif]

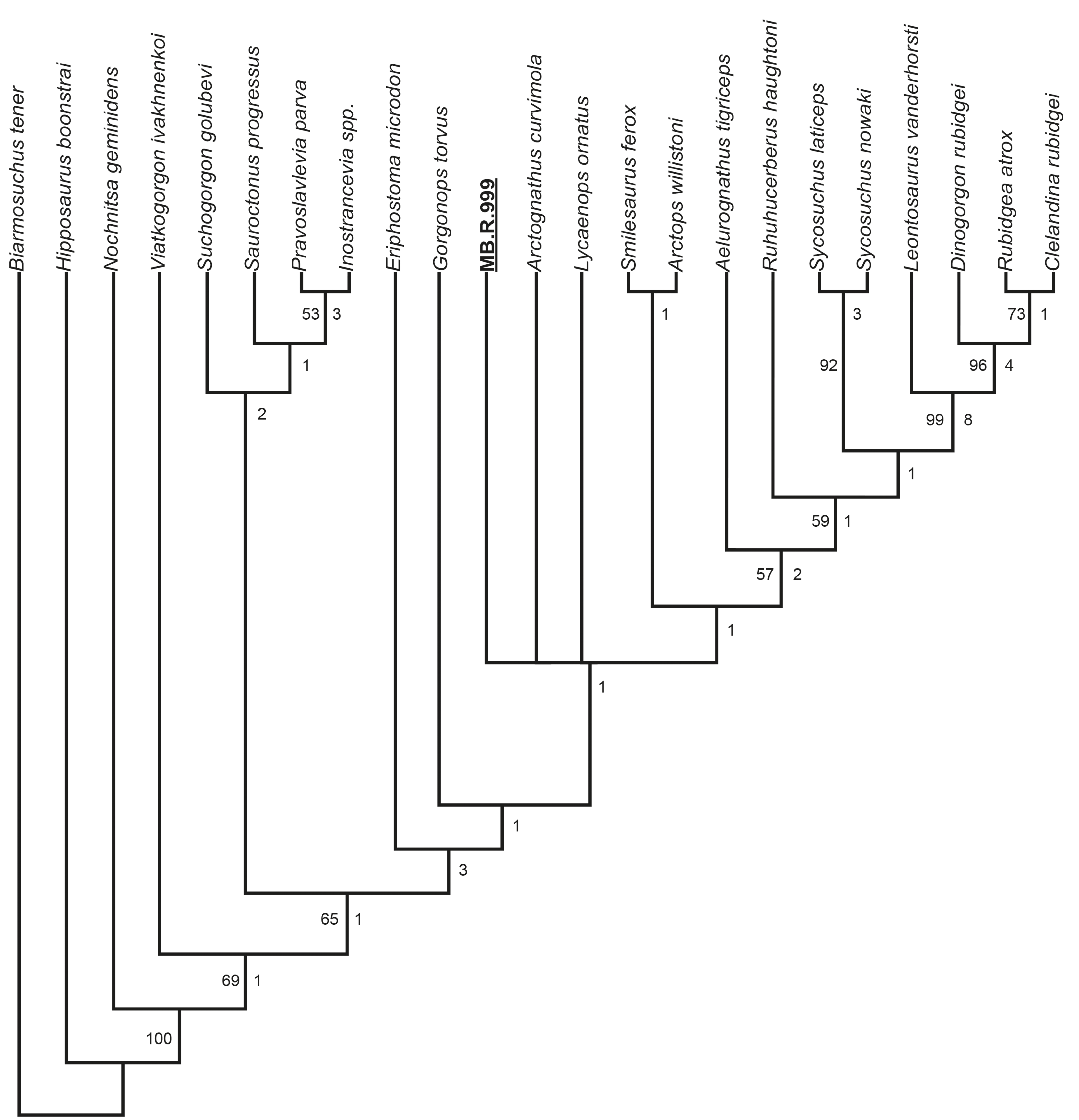

Supplement: S3 Fig — Numbers left under nodes indicate bootstrapping support values above 50%. Numbers right under nodes show Bremer support indices. (TIF) [file pone.0207367.s003.tif]
