# Supplementary material for: Cranial anatomy of the gorgonopsian Cynariops robustus based on CT-reconstruction
Source: PLoS One. 2018 Nov 28;13(11):e0207367. doi: 10.1371/journal.pone.0207367 (PMC6261584; doi:10.1371/journal.pone.0207367)
Supplement: S2 File — For reprint [22] and use of Figs 1–3 under a CC BY license, original copyright 2016 (PDF) [file pone.0207367.s005.pdf]

To whom it may concern,

I hereby grant permission to use my photographs of the specimen MB.R.999 (housed in the Museum für Naturkunde, Berlin, Germany) under the Creative Commons Attribution License CC BY 4.0 in a Plos One paper. I am aware that this license allows unrestricted use and distribution, even commercially, by third parties.

Sincerely,

Carola Radke, Berlin 25.07.2018
